# Supplementary material for: Global Epidemiology and Burden of Elderly-Onset Inflammatory Bowel Disease: A Decade in Review
Source: J Clin Med. 2023 Aug 6;12(15):5142. doi: 10.3390/jcm12155142 (PMC10420121; doi:10.3390/jcm12155142)
Supplement: Supplementary file 1 [file jcm-12-05142-s001.zip › jcm-2470604-supplementary.pdf]

Supplementary Material Table S1. Sociodemographic Index values for all estimated GBD 2019 locations, 2010-2019.

**Location**	**2010**	**2011**	**2012**	**2013**	**2014**	**2015**	**2016**	**2017**	**2018**	**2019**
Global	0.607	0.612	0.616	0.621	0.626	0.631	0.635	0.641	0.647	0.651
Central Europe, eastern Europe, and central Asia	0.732	0.735	0.738	0.742	0.745	0.748	0.751	0.754	0.758	0.76
Central Asia	0.622	0.627	0.632	0.637	0.642	0.647	0.651	0.655	0.659	0.663
Armenia	0.658	0.662	0.666	0.67	0.673	0.676	0.679	0.682	0.686	0.689
Azerbaijan	0.637	0.645	0.652	0.658	0.664	0.669	0.673	0.677	0.68	0.683
Georgia	0.665	0.668	0.672	0.676	0.68	0.684	0.688	0.693	0.697	0.702
Kazakhstan	0.688	0.692	0.696	0.7	0.704	0.708	0.712	0.716	0.72	0.723
Kyrgyzstan	0.56	0.563	0.565	0.569	0.574	0.578	0.583	0.588	0.592	0.596
Mongolia	0.566	0.57	0.575	0.579	0.584	0.588	0.592	0.597	0.601	0.606
Tajikistan	0.495	0.5	0.505	0.511	0.516	0.521	0.526	0.531	0.535	0.539
Turkmenistan	0.616	0.622	0.628	0.635	0.642	0.648	0.654	0.66	0.666	0.67
Uzbekistan	0.578	0.584	0.59	0.597	0.603	0.609	0.616	0.622	0.627	0.631
Central Europe	0.756	0.76	0.764	0.768	0.771	0.775	0.778	0.781	0.785	0.788
Albania	0.636	0.64	0.645	0.651	0.658	0.664	0.669	0.674	0.678	0.681
Bosnia and Herzegovina	0.682	0.686	0.691	0.695	0.698	0.702	0.706	0.71	0.714	0.718
Bulgaria	0.733	0.737	0.74	0.743	0.746	0.75	0.752	0.755	0.76	0.764
Croatia	0.763	0.767	0.77	0.774	0.777	0.781	0.784	0.788	0.791	0.794
Czech Republic	0.813	0.816	0.818	0.819	0.82	0.82	0.82	0.822	0.825	0.828
Hungary	0.772	0.773	0.774	0.774	0.775	0.778	0.781	0.784	0.788	0.791
Montenegro	0.754	0.759	0.764	0.768	0.773	0.777	0.78	0.784	0.788	0.791
North Macedonia	0.709	0.713	0.717	0.722	0.726	0.73	0.734	0.738	0.741	0.744
Poland	0.763	0.77	0.775	0.78	0.784	0.788	0.791	0.795	0.798	0.802
Romania	0.726	0.729	0.734	0.74	0.741	0.744	0.747	0.752	0.756	0.76
Serbia	0.729	0.735	0.739	0.744	0.748	0.753	0.756	0.76	0.763	0.767
Slovakia	0.789	0.794	0.798	0.801	0.803	0.803	0.804	0.805	0.808	0.812
Slovenia	0.822	0.824	0.825	0.827	0.829	0.831	0.833	0.835	0.838	0.84
Eastern Europe	0.762	0.765	0.768	0.772	0.777	0.781	0.785	0.788	0.791	0.793
Belarus	0.703	0.709	0.713	0.719	0.725	0.73	0.734	0.738	0.742	0.745
Estonia	0.798	0.804	0.809	0.813	0.817	0.821	0.825	0.829	0.833	0.835
Latvia	0.797	0.798	0.801	0.803	0.804	0.805	0.809	0.813	0.817	0.82
Lithuania	0.797	0.801	0.808	0.813	0.817	0.822	0.829	0.835	0.839	0.843
Moldova	0.644	0.651	0.658	0.665	0.672	0.677	0.683	0.688	0.693	0.696
Russia	0.775	0.777	0.779	0.784	0.788	0.793	0.797	0.801	0.803	0.805
Ukraine	0.713	0.718	0.721	0.725	0.727	0.729	0.73	0.732	0.734	0.736
High income	0.82	0.823	0.826	0.829	0.832	0.835	0.839	0.842	0.845	0.847
Australasia	0.81	0.812	0.816	0.821	0.825	0.828	0.832	0.835	0.837	0.84
Australia	0.809	0.812	0.815	0.82	0.824	0.828	0.832	0.834	0.837	0.839
New Zealand	0.809	0.812	0.816	0.821	0.825	0.828	0.832	0.835	0.838	0.84
High-income Asia Pacific	0.847	0.85	0.853	0.856	0.859	0.862	0.865	0.868	0.871	0.873
Brunei	0.789	0.793	0.797	0.801	0.806	0.809	0.813	0.817	0.82	0.823
Japan	0.848	0.85	0.853	0.855	0.857	0.86	0.862	0.865	0.867	0.87
Aichi	0.859	0.861	0.864	0.866	0.869	0.871	0.874	0.876	0.879	0.881

Akita	0.806	0.808	0.811	0.813	0.816	0.819	0.822	0.824	0.827	0.83
Aomori	0.8	0.803	0.806	0.809	0.811	0.814	0.817	0.82	0.823	0.826
Chiba	0.842	0.844	0.846	0.848	0.85	0.852	0.855	0.857	0.859	0.861
Ehime	0.818	0.82	0.822	0.824	0.827	0.83	0.833	0.836	0.838	0.841
Fukui	0.833	0.836	0.838	0.84	0.842	0.844	0.846	0.849	0.851	0.854
Fukuoka	0.837	0.84	0.842	0.844	0.846	0.848	0.85	0.853	0.855	0.858
Fukushima	0.812	0.814	0.816	0.818	0.821	0.823	0.826	0.83	0.833	0.836
Gifu	0.831	0.833	0.836	0.838	0.84	0.842	0.845	0.847	0.849	0.852
Gunma	0.833	0.836	0.838	0.841	0.844	0.847	0.85	0.852	0.855	0.858
Hiroshima	0.843	0.846	0.849	0.851	0.854	0.857	0.859	0.862	0.865	0.868
Hokkaidō	0.823	0.825	0.827	0.829	0.831	0.833	0.836	0.838	0.841	0.843
Hyōgo	0.845	0.848	0.85	0.852	0.854	0.857	0.859	0.862	0.864	0.866
Ibaraki	0.834	0.837	0.839	0.842	0.844	0.847	0.85	0.852	0.855	0.858
Ishikawa	0.835	0.837	0.839	0.841	0.843	0.846	0.848	0.851	0.854	0.856
Iwate	0.803	0.806	0.808	0.811	0.814	0.818	0.821	0.824	0.828	0.831
Kagawa	0.831	0.834	0.837	0.839	0.842	0.844	0.847	0.85	0.853	0.855
Kagoshima	0.806	0.808	0.811	0.813	0.816	0.819	0.822	0.825	0.828	0.831
Kanagawa	0.867	0.869	0.87	0.872	0.873	0.875	0.877	0.878	0.88	0.882
Kōchi	0.807	0.81	0.812	0.815	0.817	0.82	0.823	0.826	0.829	0.832
Kumamoto	0.808	0.811	0.813	0.816	0.819	0.822	0.825	0.828	0.831	0.834
Kyōto	0.855	0.857	0.859	0.861	0.863	0.865	0.868	0.87	0.872	0.875
Mie	0.832	0.835	0.838	0.841	0.844	0.847	0.85	0.853	0.856	0.859
Miyagi	0.831	0.834	0.837	0.839	0.842	0.845	0.848	0.851	0.854	0.857
Miyazaki	0.799	0.802	0.805	0.807	0.81	0.814	0.817	0.82	0.823	0.826
Nagano	0.835	0.837	0.839	0.841	0.843	0.845	0.848	0.851	0.853	0.856
Nagasaki	0.801	0.804	0.807	0.81	0.813	0.815	0.818	0.821	0.824	0.827
Nara	0.834	0.836	0.837	0.839	0.841	0.843	0.845	0.847	0.849	0.851
Niigata	0.821	0.823	0.826	0.829	0.832	0.834	0.837	0.84	0.842	0.845
Ōita	0.825	0.827	0.83	0.832	0.834	0.836	0.839	0.841	0.844	0.847
Okayama	0.838	0.841	0.843	0.845	0.848	0.85	0.853	0.855	0.858	0.861
Okinawa	0.795	0.798	0.8	0.802	0.804	0.807	0.81	0.813	0.816	0.819
Ōsaka	0.856	0.858	0.86	0.862	0.864	0.866	0.869	0.871	0.873	0.875
Saga	0.812	0.814	0.816	0.818	0.82	0.822	0.825	0.828	0.831	0.834
Saitama	0.837	0.839	0.841	0.843	0.845	0.847	0.849	0.851	0.854	0.856
Shiga	0.853	0.855	0.858	0.86	0.862	0.864	0.867	0.869	0.872	0.874
Shimane	0.809	0.812	0.815	0.818	0.821	0.824	0.827	0.83	0.833	0.836
Shizuoka	0.84	0.843	0.845	0.848	0.851	0.853	0.856	0.858	0.861	0.864
Tochigi	0.834	0.836	0.839	0.841	0.844	0.847	0.851	0.854	0.857	0.859
Tokushima	0.828	0.831	0.833	0.836	0.839	0.842	0.845	0.848	0.851	0.854
Tōkyō	0.913	0.915	0.917	0.918	0.92	0.921	0.923	0.924	0.925	0.927
Tottori	0.815	0.816	0.818	0.82	0.822	0.825	0.827	0.83	0.833	0.835
Toyama	0.842	0.844	0.846	0.848	0.85	0.853	0.855	0.858	0.861	0.863
Wakayama	0.819	0.822	0.825	0.828	0.832	0.835	0.838	0.842	0.845	0.848
Yamagata	0.81	0.813	0.816	0.818	0.821	0.824	0.827	0.83	0.833	0.835
Yamaguchi	0.83	0.833	0.836	0.839	0.841	0.844	0.847	0.849	0.852	0.855
Yamanashi	0.836	0.839	0.841	0.843	0.846	0.848	0.851	0.853	0.856	0.858
South Korea	0.842	0.846	0.851	0.855	0.859	0.863	0.867	0.871	0.875	0.878

Singapore	0.835	0.839	0.843	0.847	0.85	0.852	0.855	0.858	0.86	0.861
High-income North America	0.834	0.837	0.841	0.844	0.847	0.85	0.854	0.857	0.859	0.86
Canada	0.851	0.853	0.856	0.859	0.861	0.864	0.867	0.869	0.871	0.873
Greenland	0.728	0.734	0.737	0.74	0.743	0.747	0.751	0.756	0.759	0.761
USA	0.832	0.835	0.839	0.842	0.845	0.849	0.853	0.856	0.858	0.859
Alabama	0.801	0.805	0.809	0.811	0.812	0.814	0.816	0.818	0.819	0.819
Alaska	0.817	0.821	0.826	0.83	0.834	0.839	0.844	0.848	0.85	0.851
Arizona	0.81	0.814	0.818	0.822	0.825	0.829	0.834	0.838	0.84	0.842
Arkansas	0.783	0.787	0.79	0.794	0.796	0.799	0.803	0.807	0.809	0.811
California	0.838	0.841	0.845	0.848	0.852	0.856	0.861	0.865	0.868	0.87
Colorado	0.847	0.851	0.855	0.859	0.863	0.867	0.871	0.874	0.876	0.877
Connecticut	0.881	0.884	0.887	0.89	0.893	0.896	0.899	0.901	0.902	0.902
Delaware	0.843	0.847	0.85	0.852	0.854	0.857	0.859	0.861	0.862	0.863
Washington, DC	0.876	0.877	0.879	0.88	0.881	0.883	0.884	0.886	0.886	0.886
Florida	0.832	0.836	0.839	0.842	0.844	0.846	0.85	0.852	0.854	0.856
Georgia	0.812	0.817	0.821	0.825	0.828	0.832	0.835	0.838	0.84	0.841
Hawaii	0.836	0.84	0.843	0.847	0.85	0.853	0.856	0.858	0.859	0.86
Idaho	0.807	0.81	0.813	0.815	0.817	0.82	0.823	0.825	0.827	0.827
Illinois	0.848	0.851	0.854	0.858	0.86	0.863	0.867	0.869	0.871	0.872
Indiana	0.812	0.816	0.819	0.823	0.826	0.829	0.833	0.835	0.837	0.838
Iowa	0.833	0.836	0.84	0.843	0.847	0.851	0.856	0.859	0.862	0.864
Kansas	0.822	0.826	0.83	0.835	0.84	0.845	0.85	0.854	0.857	0.858
Kentucky	0.79	0.794	0.797	0.8	0.803	0.806	0.81	0.812	0.814	0.815
Louisiana	0.794	0.798	0.801	0.805	0.808	0.812	0.817	0.82	0.822	0.823
Maine	0.84	0.842	0.845	0.848	0.85	0.853	0.856	0.858	0.86	0.862
Maryland	0.867	0.87	0.873	0.876	0.878	0.881	0.884	0.885	0.886	0.887
Massachusetts	0.889	0.892	0.894	0.897	0.899	0.902	0.904	0.906	0.907	0.907
Michigan	0.836	0.838	0.84	0.843	0.845	0.849	0.854	0.858	0.861	0.863
Minnesota	0.86	0.863	0.866	0.87	0.873	0.876	0.879	0.882	0.884	0.886
Mississippi	0.774	0.779	0.784	0.789	0.792	0.796	0.8	0.803	0.804	0.805
Missouri	0.818	0.821	0.825	0.828	0.831	0.834	0.838	0.841	0.843	0.844
Montana	0.823	0.826	0.829	0.833	0.837	0.842	0.847	0.851	0.854	0.856
Nebraska	0.833	0.836	0.84	0.845	0.849	0.853	0.857	0.86	0.862	0.862
Nevada	0.822	0.825	0.827	0.829	0.829	0.83	0.832	0.834	0.834	0.835
New Hampshire	0.874	0.877	0.88	0.883	0.886	0.89	0.893	0.895	0.897	0.898
New Jersey	0.872	0.875	0.878	0.88	0.883	0.886	0.888	0.89	0.892	0.892
New Mexico	0.793	0.798	0.803	0.807	0.811	0.815	0.819	0.823	0.825	0.826
New York	0.863	0.866	0.869	0.872	0.875	0.878	0.881	0.883	0.884	0.884
North Carolina	0.813	0.817	0.822	0.826	0.829	0.832	0.835	0.836	0.837	0.838
North Dakota	0.832	0.835	0.84	0.846	0.853	0.861	0.868	0.873	0.876	0.876
Ohio	0.823	0.826	0.829	0.832	0.835	0.838	0.841	0.843	0.845	0.846
Oklahoma	0.793	0.797	0.801	0.805	0.808	0.813	0.818	0.823	0.826	0.827
Oregon	0.838	0.842	0.845	0.848	0.852	0.856	0.86	0.864	0.866	0.868
Pennsylvania	0.846	0.849	0.852	0.855	0.857	0.861	0.864	0.866	0.868	0.87
Rhode Island	0.86	0.863	0.866	0.869	0.872	0.875	0.877	0.879	0.881	0.882
South Carolina	0.807	0.811	0.816	0.82	0.823	0.826	0.828	0.83	0.831	0.832
South Dakota	0.817	0.82	0.824	0.828	0.833	0.838	0.843	0.847	0.849	0.85

Tennessee	0.795	0.8	0.805	0.81	0.815	0.82	0.823	0.826	0.827	0.827
Texas	0.794	0.799	0.803	0.808	0.812	0.818	0.824	0.83	0.833	0.835
Utah	0.818	0.822	0.827	0.831	0.834	0.839	0.843	0.847	0.849	0.851
Vermont	0.864	0.866	0.869	0.872	0.875	0.879	0.883	0.885	0.887	0.889
Virginia	0.854	0.858	0.861	0.864	0.867	0.869	0.872	0.874	0.876	0.877
Washington	0.85	0.853	0.856	0.86	0.863	0.866	0.87	0.873	0.875	0.876
West Virginia	0.783	0.786	0.789	0.793	0.797	0.802	0.806	0.809	0.811	0.812
Wisconsin	0.844	0.848	0.851	0.855	0.858	0.861	0.864	0.866	0.868	0.869
Wyoming	0.829	0.833	0.837	0.84	0.843	0.847	0.85	0.853	0.855	0.856
Southern Latin America	0.676	0.681	0.686	0.689	0.692	0.701	0.71	0.716	0.719	0.721
Argentina	0.665	0.67	0.674	0.677	0.679	0.687	0.696	0.702	0.706	0.708
Chile	0.706	0.712	0.719	0.724	0.728	0.738	0.747	0.753	0.756	0.759
Uruguay	0.653	0.658	0.663	0.668	0.673	0.678	0.684	0.688	0.693	0.697
Western Europe	0.817	0.821	0.824	0.827	0.83	0.832	0.835	0.838	0.841	0.843
Andorra	0.876	0.879	0.881	0.883	0.885	0.887	0.889	0.891	0.892	0.894
Austria	0.826	0.83	0.833	0.835	0.838	0.839	0.841	0.844	0.847	0.849
Belgium	0.82	0.824	0.829	0.834	0.837	0.841	0.843	0.846	0.849	0.851
Cyprus	0.82	0.824	0.827	0.829	0.831	0.832	0.834	0.836	0.838	0.841
Denmark	0.87	0.873	0.875	0.878	0.88	0.882	0.884	0.886	0.888	0.89
Finland	0.828	0.831	0.834	0.837	0.84	0.844	0.848	0.851	0.853	0.856
France	0.806	0.809	0.812	0.815	0.819	0.822	0.826	0.829	0.832	0.834
Germany	0.878	0.881	0.883	0.886	0.888	0.89	0.892	0.894	0.896	0.898
Greece	0.779	0.782	0.785	0.786	0.786	0.787	0.788	0.79	0.792	0.794
Iceland	0.846	0.847	0.847	0.848	0.85	0.854	0.858	0.863	0.866	0.869
Ireland	0.831	0.835	0.839	0.842	0.845	0.85	0.854	0.859	0.864	0.867
Israel	0.781	0.784	0.787	0.79	0.792	0.794	0.796	0.798	0.8	0.803
Italy	0.78	0.782	0.784	0.787	0.789	0.791	0.794	0.796	0.798	0.801
Luxembourg	0.872	0.874	0.877	0.88	0.883	0.886	0.889	0.892	0.894	0.895
Malta	0.764	0.768	0.772	0.775	0.779	0.784	0.788	0.793	0.797	0.801
Monaco	0.886	0.888	0.89	0.892	0.893	0.895	0.897	0.899	0.901	0.902
Netherlands	0.861	0.864	0.866	0.869	0.871	0.874	0.876	0.878	0.881	0.883
Norway	0.885	0.889	0.893	0.896	0.9	0.903	0.907	0.91	0.912	0.913
Portugal	0.709	0.714	0.718	0.722	0.726	0.729	0.732	0.736	0.739	0.743
San Marino	0.872	0.874	0.876	0.877	0.879	0.881	0.882	0.882	0.883	0.884
Spain	0.743	0.746	0.749	0.752	0.754	0.756	0.759	0.761	0.764	0.767
Sweden	0.849	0.852	0.855	0.858	0.86	0.863	0.865	0.868	0.87	0.872
Stockholm	0.886	0.889	0.891	0.893	0.895	0.897	0.899	0.901	0.903	0.904
Sweden except Stockholm	0.838	0.841	0.844	0.846	0.849	0.851	0.853	0.856	0.858	0.86
Switzerland	0.912	0.914	0.917	0.919	0.921	0.922	0.924	0.926	0.928	0.929
UK	0.816	0.82	0.825	0.83	0.834	0.837	0.839	0.842	0.845	0.847
England	0.82	0.823	0.828	0.833	0.837	0.84	0.842	0.845	0.848	0.85
East Midlands	0.796	0.8	0.804	0.809	0.813	0.816	0.819	0.821	0.824	0.826
Derby	0.805	0.809	0.814	0.821	0.825	0.829	0.832	0.835	0.838	0.841
Derbyshire	0.779	0.783	0.789	0.795	0.799	0.803	0.806	0.809	0.811	0.814
Leicester	0.797	0.801	0.805	0.809	0.812	0.815	0.817	0.819	0.821	0.823
Leicestershire	0.819	0.822	0.826	0.83	0.833	0.835	0.838	0.84	0.842	0.845
Lincolnshire	0.777	0.781	0.786	0.791	0.795	0.799	0.802	0.804	0.807	0.81

Northamptonshire	0.795	0.799	0.804	0.81	0.814	0.817	0.82	0.822	0.825	0.827
Nottingham	0.828	0.832	0.836	0.84	0.842	0.844	0.846	0.848	0.85	0.852
Nottinghamshire	0.781	0.784	0.789	0.794	0.797	0.8	0.803	0.806	0.809	0.811
Rutland	0.821	0.824	0.827	0.831	0.834	0.836	0.838	0.84	0.842	0.844
East of England	0.814	0.817	0.822	0.827	0.83	0.833	0.836	0.839	0.841	0.844
Bedford	0.818	0.821	0.825	0.83	0.834	0.836	0.839	0.842	0.844	0.847
Cambridgeshire	0.851	0.854	0.858	0.862	0.865	0.868	0.87	0.872	0.875	0.877
Central Bedfordshire	0.811	0.815	0.819	0.824	0.827	0.83	0.833	0.836	0.839	0.841
Essex	0.805	0.809	0.813	0.817	0.821	0.823	0.825	0.828	0.83	0.833
Hertfordshire	0.851	0.854	0.857	0.862	0.865	0.867	0.87	0.872	0.875	0.877
Luton	0.803	0.807	0.812	0.817	0.82	0.823	0.825	0.828	0.83	0.832
Norfolk	0.793	0.796	0.801	0.806	0.81	0.813	0.815	0.818	0.821	0.823
Peterborough	0.78	0.785	0.791	0.798	0.802	0.806	0.809	0.813	0.816	0.818
Southend-on-Sea	0.783	0.787	0.792	0.797	0.8	0.803	0.805	0.807	0.81	0.812
Suffolk	0.794	0.799	0.804	0.81	0.813	0.816	0.819	0.822	0.824	0.827
Thurrock	0.776	0.78	0.784	0.789	0.792	0.794	0.796	0.799	0.801	0.804
Greater London	0.871	0.874	0.878	0.883	0.886	0.888	0.89	0.892	0.894	0.895
Barking and Dagenham	0.751	0.756	0.761	0.768	0.773	0.777	0.78	0.783	0.787	0.79
Barnet	0.852	0.855	0.859	0.863	0.866	0.869	0.872	0.874	0.876	0.878
Bexley	0.799	0.803	0.807	0.812	0.817	0.82	0.824	0.827	0.83	0.833
Brent	0.824	0.827	0.832	0.837	0.84	0.842	0.844	0.846	0.848	0.851
Bromley	0.835	0.837	0.841	0.844	0.847	0.849	0.852	0.854	0.856	0.858
Camden	0.917	0.92	0.922	0.925	0.926	0.928	0.929	0.93	0.931	0.931
Croydon	0.816	0.818	0.822	0.826	0.829	0.831	0.833	0.835	0.838	0.84
Ealing	0.842	0.845	0.849	0.854	0.858	0.862	0.865	0.868	0.87	0.872
Enfield	0.806	0.81	0.815	0.82	0.823	0.826	0.829	0.831	0.834	0.836
Greenwich	0.805	0.808	0.813	0.818	0.822	0.825	0.827	0.83	0.832	0.834
Hackney	0.853	0.858	0.864	0.87	0.873	0.876	0.877	0.879	0.881	0.882
Hammersmith and Fulham	0.912	0.914	0.917	0.92	0.922	0.924	0.925	0.927	0.928	0.929
Haringey	0.832	0.835	0.84	0.845	0.848	0.851	0.854	0.856	0.858	0.86
Harrow	0.834	0.836	0.839	0.843	0.845	0.847	0.848	0.85	0.852	0.854
Havering	0.793	0.796	0.8	0.805	0.808	0.811	0.814	0.817	0.82	0.823
Hillingdon	0.856	0.859	0.863	0.868	0.871	0.874	0.877	0.879	0.881	0.882
Hounslow	0.85	0.854	0.858	0.864	0.869	0.873	0.876	0.88	0.883	0.885
Islington	0.9	0.903	0.906	0.909	0.911	0.913	0.914	0.916	0.917	0.918
Kensington and Chelsea	0.926	0.929	0.931	0.934	0.935	0.937	0.938	0.939	0.94	0.941
Kingston upon Thames	0.884	0.886	0.888	0.891	0.893	0.895	0.896	0.898	0.9	0.902
Lambeth	0.878	0.881	0.886	0.89	0.894	0.897	0.899	0.902	0.904	0.906
Lewisham	0.816	0.82	0.824	0.829	0.833	0.836	0.839	0.842	0.844	0.846
Merton	0.845	0.848	0.853	0.858	0.862	0.866	0.869	0.872	0.874	0.876
Newham	0.798	0.803	0.808	0.814	0.818	0.822	0.824	0.827	0.829	0.831
Redbridge	0.813	0.816	0.82	0.825	0.828	0.831	0.834	0.836	0.839	0.841
Richmond upon Thames	0.899	0.901	0.904	0.908	0.911	0.914	0.917	0.92	0.922	0.924
Southwark	0.887	0.891	0.895	0.899	0.902	0.904	0.906	0.908	0.909	0.91
Sutton	0.819	0.822	0.826	0.831	0.835	0.838	0.841	0.844	0.847	0.849
Tower Hamlets	0.873	0.877	0.881	0.885	0.888	0.89	0.892	0.893	0.895	0.896
Waltham Forest	0.792	0.796	0.801	0.807	0.811	0.815	0.818	0.822	0.825	0.827

Wandsworth	0.899	0.901	0.904	0.907	0.909	0.911	0.913	0.915	0.916	0.918
Westminster	0.914	0.916	0.919	0.922	0.924	0.925	0.927	0.928	0.929	0.93
North East England	0.788	0.792	0.797	0.802	0.805	0.808	0.811	0.814	0.817	0.819
County Durham	0.777	0.78	0.785	0.79	0.793	0.796	0.798	0.801	0.803	0.805
Darlington	0.791	0.796	0.801	0.807	0.812	0.815	0.818	0.821	0.824	0.826
Gateshead	0.79	0.794	0.799	0.805	0.809	0.812	0.815	0.817	0.82	0.822
Hartlepool	0.749	0.753	0.759	0.766	0.771	0.774	0.778	0.781	0.784	0.786
Middlesbroug h	0.765	0.769	0.774	0.78	0.783	0.786	0.788	0.791	0.793	0.796
Newcastle upon Tyne	0.846	0.849	0.853	0.856	0.858	0.86	0.861	0.863	0.865	0.866
North Tyneside	0.793	0.797	0.802	0.808	0.812	0.815	0.818	0.821	0.824	0.826
Northumberl and	0.784	0.787	0.792	0.797	0.801	0.804	0.807	0.809	0.812	0.814
Redcar and Cleveland	0.756	0.76	0.765	0.772	0.776	0.78	0.783	0.786	0.789	0.791
South Tyneside	0.756	0.76	0.766	0.772	0.776	0.78	0.783	0.786	0.789	0.792
Stockton-on-Tees	0.791	0.795	0.8	0.805	0.809	0.812	0.815	0.817	0.82	0.822
Sunderland	0.777	0.781	0.786	0.792	0.796	0.799	0.802	0.805	0.807	0.81
North West England	0.8	0.804	0.809	0.815	0.819	0.822	0.825	0.827	0.83	0.832
Blackburn with Darwen	0.755	0.761	0.767	0.775	0.78	0.785	0.789	0.792	0.795	0.798
Blackpool	0.737	0.741	0.746	0.753	0.757	0.76	0.763	0.766	0.769	0.772
Bolton	0.766	0.77	0.775	0.782	0.786	0.789	0.792	0.795	0.797	0.8
Bury	0.784	0.788	0.793	0.8	0.804	0.808	0.811	0.814	0.817	0.819
Cheshire East	0.838	0.842	0.847	0.852	0.856	0.86	0.863	0.865	0.868	0.87
Cheshire West and Chester	0.834	0.837	0.842	0.847	0.85	0.853	0.855	0.858	0.86	0.862
Cumbria	0.793	0.797	0.803	0.81	0.815	0.819	0.822	0.825	0.828	0.83
Halton	0.778	0.783	0.789	0.797	0.802	0.806	0.81	0.813	0.817	0.819
Knowsley	0.762	0.766	0.772	0.779	0.783	0.787	0.79	0.794	0.797	0.8
Lancashire	0.801	0.805	0.81	0.815	0.819	0.822	0.824	0.827	0.829	0.831
Liverpool	0.821	0.824	0.827	0.831	0.833	0.835	0.836	0.838	0.84	0.842
Manchester	0.848	0.852	0.856	0.86	0.863	0.865	0.867	0.869	0.872	0.873
Oldham	0.744	0.749	0.755	0.762	0.766	0.77	0.773	0.776	0.779	0.781
Rochdale	0.753	0.757	0.763	0.77	0.774	0.778	0.781	0.784	0.787	0.79
Salford	0.793	0.798	0.803	0.81	0.814	0.817	0.82	0.822	0.825	0.827
Sefton	0.789	0.792	0.795	0.799	0.802	0.804	0.806	0.808	0.81	0.812
St Helens	0.765	0.769	0.774	0.78	0.784	0.787	0.789	0.792	0.794	0.797
Stockport	0.82	0.824	0.828	0.834	0.837	0.84	0.843	0.845	0.848	0.85
Tameside	0.757	0.76	0.766	0.772	0.776	0.779	0.782	0.785	0.788	0.79
Trafford	0.851	0.854	0.859	0.864	0.867	0.871	0.874	0.876	0.879	0.881
Warrington	0.83	0.834	0.84	0.846	0.85	0.854	0.858	0.861	0.864	0.866
Wigan	0.759	0.763	0.769	0.775	0.779	0.782	0.785	0.788	0.791	0.793
Wirral	0.774	0.777	0.783	0.788	0.792	0.796	0.798	0.801	0.804	0.806
South East England	0.838	0.841	0.845	0.85	0.853	0.855	0.858	0.86	0.862	0.864
Bracknell Forest	0.849	0.852	0.857	0.861	0.865	0.868	0.871	0.873	0.876	0.878
Brighton and Hove	0.871	0.873	0.876	0.879	0.881	0.883	0.885	0.887	0.889	0.891
Buckinghams hire	0.853	0.856	0.86	0.864	0.866	0.869	0.871	0.873	0.875	0.877
East Sussex	0.795	0.799	0.803	0.809	0.812	0.816	0.819	0.822	0.825	0.827
Hampshire	0.833	0.836	0.841	0.846	0.85	0.853	0.855	0.858	0.86	0.862
Isle of Wight	0.778	0.781	0.785	0.79	0.793	0.796	0.798	0.8	0.803	0.805
Kent	0.805	0.808	0.813	0.818	0.821	0.824	0.826	0.828	0.831	0.833

Medway	0.78	0.783	0.788	0.792	0.796	0.798	0.8	0.802	0.805	0.807
Milton Keynes	0.834	0.838	0.844	0.85	0.855	0.859	0.862	0.866	0.869	0.87
Oxfordshire	0.864	0.867	0.871	0.875	0.878	0.881	0.883	0.886	0.888	0.89
Portsmouth	0.836	0.839	0.842	0.846	0.849	0.85	0.852	0.854	0.856	0.858
Reading	0.878	0.88	0.883	0.886	0.889	0.89	0.891	0.893	0.894	0.896
Slough	0.831	0.835	0.841	0.847	0.851	0.854	0.857	0.86	0.862	0.864
Southampton	0.837	0.839	0.841	0.844	0.846	0.848	0.849	0.851	0.853	0.855
Surrey	0.871	0.874	0.877	0.881	0.884	0.886	0.889	0.891	0.893	0.895
West Berkshire	0.857	0.861	0.865	0.87	0.874	0.876	0.878	0.88	0.882	0.883
West Sussex	0.825	0.829	0.833	0.838	0.841	0.844	0.847	0.849	0.852	0.854
Windsor and Maidenhead	0.877	0.88	0.884	0.889	0.892	0.895	0.898	0.9	0.903	0.905
Wokingham	0.883	0.885	0.887	0.891	0.893	0.896	0.898	0.9	0.902	0.904
South West England	0.819	0.823	0.827	0.832	0.835	0.838	0.841	0.843	0.846	0.848
Bath and North East Somerset	0.865	0.868	0.871	0.874	0.876	0.878	0.88	0.882	0.884	0.886
Bournemouth	0.838	0.84	0.844	0.847	0.85	0.852	0.854	0.856	0.858	0.861
Bristol, City of	0.861	0.864	0.868	0.872	0.875	0.877	0.88	0.882	0.884	0.886
Cornwall	0.796	0.799	0.804	0.809	0.812	0.815	0.818	0.82	0.823	0.825
Devon	0.817	0.821	0.825	0.829	0.832	0.835	0.837	0.839	0.841	0.843
Dorset	0.805	0.808	0.813	0.818	0.822	0.825	0.827	0.83	0.833	0.835
Gloucestershire	0.829	0.833	0.837	0.842	0.846	0.849	0.852	0.855	0.857	0.859
North Somerset	0.81	0.814	0.819	0.825	0.829	0.833	0.836	0.838	0.841	0.843
Plymouth	0.809	0.812	0.816	0.821	0.824	0.826	0.828	0.83	0.832	0.834
Poole	0.819	0.823	0.828	0.833	0.837	0.84	0.843	0.846	0.848	0.85
Somerset	0.794	0.798	0.802	0.808	0.812	0.815	0.818	0.821	0.824	0.826
South Gloucestershire	0.843	0.847	0.851	0.856	0.86	0.863	0.865	0.868	0.871	0.873
Swindon	0.821	0.825	0.83	0.835	0.839	0.842	0.844	0.846	0.849	0.85
Torbay	0.765	0.768	0.772	0.777	0.78	0.782	0.785	0.787	0.79	0.793
Wiltshire	0.811	0.815	0.82	0.825	0.829	0.833	0.836	0.839	0.841	0.844
West Midlands	0.792	0.796	0.801	0.807	0.811	0.814	0.817	0.82	0.823	0.825
Birmingham	0.797	0.801	0.806	0.811	0.815	0.818	0.82	0.823	0.825	0.827
Coventry	0.81	0.813	0.818	0.823	0.826	0.829	0.832	0.835	0.837	0.84
Dudley	0.76	0.763	0.768	0.773	0.777	0.779	0.782	0.785	0.788	0.79
Herefordshire, County of	0.796	0.8	0.805	0.811	0.816	0.819	0.823	0.826	0.829	0.832
Sandwell	0.741	0.746	0.752	0.759	0.764	0.768	0.772	0.775	0.778	0.781
Shropshire	0.802	0.806	0.81	0.815	0.819	0.822	0.825	0.827	0.83	0.832
Solihull	0.827	0.83	0.834	0.839	0.842	0.846	0.849	0.852	0.855	0.858
Staffordshire	0.793	0.797	0.801	0.805	0.808	0.811	0.813	0.815	0.817	0.819
Stoke-on-Trent	0.745	0.75	0.756	0.764	0.769	0.773	0.776	0.78	0.783	0.786
Telford and Wrekin	0.778	0.782	0.787	0.794	0.798	0.801	0.804	0.807	0.81	0.813
Walsall	0.74	0.745	0.751	0.758	0.762	0.766	0.77	0.773	0.776	0.779
Warwickshire	0.825	0.829	0.834	0.839	0.843	0.846	0.849	0.852	0.855	0.857
Wolverhampton	0.764	0.768	0.774	0.78	0.784	0.787	0.79	0.793	0.796	0.799
Worcestershire	0.802	0.805	0.809	0.814	0.818	0.82	0.823	0.826	0.828	0.831
Yorkshire and the Humber	0.793	0.797	0.802	0.808	0.812	0.815	0.818	0.821	0.823	0.826
Barnsley	0.737	0.741	0.746	0.753	0.758	0.761	0.765	0.768	0.771	0.774
Bradford	0.761	0.766	0.773	0.78	0.784	0.788	0.791	0.794	0.797	0.8

Calderdale	0.783	0.788	0.794	0.802	0.806	0.81	0.813	0.816	0.819	0.821
Doncaster	0.743	0.748	0.754	0.761	0.766	0.77	0.774	0.777	0.781	0.783
East Riding of Yorkshire	0.794	0.798	0.802	0.806	0.81	0.812	0.814	0.817	0.819	0.821
Kingston upon Hull, City of	0.764	0.768	0.772	0.778	0.781	0.783	0.785	0.787	0.79	0.792
Kirklees	0.777	0.782	0.787	0.793	0.797	0.8	0.803	0.805	0.808	0.81
Leeds	0.838	0.842	0.845	0.849	0.853	0.855	0.858	0.86	0.862	0.864
North East Lincolnshire	0.746	0.751	0.757	0.764	0.768	0.771	0.774	0.778	0.781	0.784
North Lincolnshire	0.781	0.785	0.79	0.796	0.799	0.8	0.801	0.803	0.805	0.806
North Yorkshire	0.811	0.815	0.819	0.824	0.827	0.83	0.832	0.834	0.836	0.838
Rotherham	0.75	0.754	0.76	0.766	0.77	0.773	0.775	0.778	0.781	0.784
Sheffield	0.822	0.826	0.83	0.834	0.837	0.84	0.842	0.844	0.847	0.848
Wakefield	0.755	0.76	0.765	0.772	0.777	0.78	0.783	0.786	0.789	0.792
York	0.863	0.865	0.868	0.871	0.873	0.875	0.877	0.879	0.881	0.883
Northern Ireland	0.792	0.796	0.802	0.808	0.811	0.815	0.818	0.821	0.823	0.825
Scotland	0.805	0.809	0.814	0.819	0.823	0.826	0.828	0.831	0.833	0.834
Wales	0.788	0.792	0.797	0.802	0.806	0.809	0.812	0.815	0.818	0.82
Latin America and Caribbean	0.589	0.594	0.6	0.605	0.611	0.616	0.62	0.625	0.629	0.633
Andean Latin America	0.585	0.591	0.596	0.602	0.608	0.613	0.618	0.624	0.628	0.632
Bolivia	0.521	0.525	0.528	0.533	0.538	0.544	0.55	0.556	0.562	0.566
Ecuador	0.591	0.597	0.603	0.609	0.615	0.621	0.626	0.632	0.636	0.64
Peru	0.601	0.608	0.614	0.62	0.625	0.63	0.635	0.64	0.645	0.648
Caribbean	0.598	0.601	0.605	0.609	0.612	0.616	0.621	0.625	0.628	0.631
Antigua and Barbuda	0.709	0.713	0.716	0.719	0.723	0.727	0.731	0.735	0.739	0.743
The Bahamas	0.774	0.779	0.78	0.782	0.784	0.786	0.789	0.791	0.794	0.796
Barbados	0.718	0.721	0.725	0.728	0.73	0.733	0.735	0.737	0.74	0.742
Belize	0.564	0.569	0.574	0.579	0.583	0.588	0.592	0.596	0.6	0.603
Bermuda	0.785	0.79	0.795	0.799	0.802	0.805	0.807	0.809	0.811	0.813
Cuba	0.62	0.624	0.631	0.636	0.64	0.645	0.653	0.66	0.665	0.668
Dominica	0.7	0.705	0.709	0.713	0.717	0.721	0.724	0.727	0.728	0.729
Dominican Republic	0.544	0.549	0.554	0.559	0.563	0.569	0.574	0.58	0.587	0.592
Grenada	0.628	0.633	0.637	0.641	0.645	0.65	0.654	0.659	0.664	0.669
Guyana	0.565	0.571	0.577	0.583	0.59	0.596	0.602	0.608	0.614	0.618
Haiti	0.395	0.399	0.403	0.407	0.412	0.416	0.42	0.424	0.428	0.432
Jamaica	0.653	0.657	0.661	0.664	0.668	0.671	0.675	0.678	0.681	0.684
Puerto Rico	0.769	0.774	0.779	0.785	0.793	0.802	0.808	0.811	0.813	0.814
Saint Kitts and Nevis	0.706	0.71	0.714	0.719	0.724	0.728	0.733	0.738	0.742	0.746
Saint Lucia	0.634	0.639	0.643	0.648	0.652	0.656	0.659	0.663	0.667	0.67
Saint Vincent and the Grenadines	0.589	0.593	0.598	0.602	0.606	0.61	0.615	0.619	0.623	0.627
Suriname	0.598	0.602	0.607	0.611	0.616	0.62	0.625	0.629	0.633	0.636
Trinidad and Tobago	0.732	0.736	0.739	0.742	0.745	0.748	0.751	0.753	0.755	0.757
Virgin Islands	0.785	0.788	0.79	0.791	0.792	0.794	0.795	0.796	0.798	0.799
Central Latin America	0.584	0.589	0.594	0.599	0.604	0.609	0.614	0.618	0.623	0.626
Colombia	0.574	0.582	0.589	0.596	0.603	0.609	0.616	0.622	0.628	0.633
Costa Rica	0.637	0.642	0.647	0.652	0.657	0.662	0.667	0.672	0.676	0.68
El Salvador	0.526	0.531	0.536	0.542	0.547	0.552	0.558	0.563	0.568	0.573
Guatemala	0.472	0.478	0.485	0.491	0.498	0.504	0.51	0.516	0.522	0.526

Honduras	0.454	0.459	0.464	0.469	0.473	0.478	0.482	0.487	0.492	0.496
Mexico	0.608	0.613	0.617	0.621	0.626	0.631	0.636	0.64	0.645	0.649
Aguascalientes	0.633	0.637	0.641	0.646	0.65	0.655	0.66	0.664	0.669	0.673
Baja California	0.653	0.656	0.66	0.663	0.667	0.671	0.675	0.68	0.685	0.688
Baja California Sur	0.653	0.658	0.662	0.666	0.67	0.675	0.68	0.684	0.689	0.693
Campeche	0.6	0.606	0.611	0.617	0.623	0.629	0.634	0.639	0.644	0.648
Chiapas	0.517	0.522	0.527	0.532	0.536	0.541	0.545	0.55	0.554	0.557
Chihuahua	0.617	0.62	0.623	0.627	0.632	0.637	0.643	0.648	0.654	0.659
Coahuila	0.625	0.628	0.631	0.634	0.639	0.644	0.648	0.653	0.658	0.663
Colima	0.642	0.646	0.65	0.654	0.659	0.664	0.669	0.673	0.678	0.682
Durango	0.571	0.575	0.58	0.585	0.591	0.596	0.602	0.608	0.614	0.618
Guanajuato	0.582	0.588	0.594	0.6	0.606	0.612	0.618	0.624	0.63	0.634
Guerrero	0.522	0.529	0.535	0.541	0.548	0.553	0.558	0.563	0.568	0.572
Hidalgo	0.559	0.565	0.571	0.577	0.584	0.59	0.596	0.601	0.606	0.611
Jalisco	0.625	0.629	0.634	0.638	0.643	0.647	0.652	0.657	0.661	0.665
México	0.625	0.629	0.632	0.636	0.64	0.644	0.648	0.652	0.657	0.66
Mexico City	0.697	0.7	0.704	0.708	0.712	0.716	0.72	0.724	0.729	0.732
Michoacán de Ocampo	0.568	0.572	0.576	0.581	0.585	0.59	0.594	0.598	0.602	0.606
Morelos	0.617	0.621	0.624	0.628	0.632	0.637	0.641	0.646	0.65	0.654
Nayarit	0.603	0.607	0.612	0.617	0.621	0.626	0.63	0.635	0.639	0.643
Nuevo León	0.661	0.665	0.669	0.673	0.677	0.682	0.686	0.691	0.696	0.699
Oaxaca	0.528	0.534	0.54	0.545	0.55	0.555	0.561	0.566	0.571	0.574
Puebla	0.567	0.572	0.577	0.583	0.588	0.593	0.598	0.603	0.608	0.612
Querétaro	0.63	0.634	0.639	0.644	0.649	0.653	0.658	0.663	0.667	0.671
Quintana Roo	0.629	0.634	0.638	0.643	0.647	0.652	0.657	0.662	0.666	0.67
San Luis Potosí	0.59	0.596	0.602	0.607	0.612	0.617	0.622	0.626	0.631	0.635
Sinaloa	0.621	0.626	0.631	0.636	0.641	0.647	0.652	0.657	0.662	0.667
Sonora	0.645	0.649	0.653	0.658	0.662	0.668	0.673	0.678	0.683	0.687
Tabasco	0.592	0.597	0.602	0.608	0.613	0.619	0.624	0.629	0.634	0.638
Tamaulipas	0.624	0.628	0.632	0.636	0.641	0.647	0.652	0.657	0.662	0.666
Tlaxcala	0.601	0.605	0.61	0.614	0.619	0.623	0.628	0.632	0.636	0.64
Veracruz de Ignacio de la Llave	0.567	0.572	0.578	0.582	0.587	0.592	0.596	0.601	0.605	0.609
Yucatán	0.594	0.599	0.605	0.611	0.616	0.622	0.627	0.631	0.636	0.64
Zacatecas	0.587	0.591	0.595	0.599	0.604	0.609	0.615	0.62	0.625	0.63
Nicaragua	0.47	0.474	0.479	0.484	0.489	0.495	0.5	0.506	0.512	0.517
Panama	0.627	0.63	0.635	0.642	0.65	0.658	0.666	0.674	0.68	0.686
Venezuela	0.586	0.591	0.596	0.6	0.604	0.607	0.608	0.608	0.608	0.607
Tropical Latin America	0.59	0.597	0.604	0.61	0.617	0.622	0.627	0.632	0.636	0.64
Brazil	0.59	0.597	0.603	0.61	0.616	0.622	0.627	0.632	0.636	0.64
Acre	0.501	0.509	0.518	0.526	0.533	0.54	0.547	0.552	0.558	0.562
Alagoas	0.461	0.469	0.477	0.485	0.492	0.498	0.504	0.509	0.514	0.518
Amapá	0.594	0.6	0.606	0.612	0.618	0.624	0.629	0.633	0.637	0.641
Amazonas	0.548	0.555	0.562	0.569	0.576	0.582	0.588	0.593	0.598	0.602
Bahia	0.505	0.514	0.521	0.529	0.536	0.542	0.548	0.553	0.558	0.562
Ceará	0.501	0.51	0.518	0.525	0.533	0.539	0.544	0.549	0.554	0.558
Distrito Federal	0.732	0.739	0.745	0.751	0.756	0.761	0.766	0.77	0.774	0.777
Espírito Santo	0.607	0.614	0.622	0.629	0.636	0.642	0.647	0.652	0.657	0.66

Goiás	0.573	0.581	0.588	0.596	0.603	0.609	0.614	0.619	0.624	0.628
Maranhão	0.376	0.385	0.394	0.403	0.412	0.42	0.427	0.433	0.439	0.444
Mato Grosso	0.587	0.595	0.603	0.61	0.617	0.623	0.629	0.634	0.638	0.642
Mato Grosso do Sul	0.585	0.593	0.601	0.608	0.615	0.621	0.626	0.631	0.636	0.639
Minas Gerais	0.596	0.603	0.61	0.616	0.622	0.627	0.632	0.636	0.64	0.643
Pará	0.51	0.518	0.526	0.534	0.542	0.549	0.554	0.56	0.565	0.569
Paraíba	0.49	0.499	0.507	0.515	0.522	0.528	0.534	0.539	0.544	0.548
Paraná	0.615	0.621	0.628	0.634	0.64	0.645	0.65	0.654	0.659	0.662
Pernambuco	0.51	0.519	0.527	0.536	0.543	0.55	0.556	0.561	0.567	0.571
Piauí	0.448	0.457	0.466	0.474	0.482	0.489	0.494	0.5	0.505	0.509
Rio de Janeiro	0.658	0.664	0.67	0.675	0.681	0.686	0.69	0.694	0.698	0.702
Rio Grande do Norte	0.519	0.527	0.535	0.543	0.551	0.557	0.562	0.567	0.572	0.576
Rio Grande do Sul	0.642	0.648	0.653	0.659	0.664	0.669	0.673	0.677	0.681	0.684
Rondônia	0.547	0.556	0.565	0.573	0.58	0.587	0.592	0.598	0.603	0.606
Roraima	0.55	0.558	0.566	0.575	0.582	0.589	0.595	0.601	0.606	0.61
Santa Catarina	0.646	0.652	0.659	0.665	0.67	0.676	0.68	0.684	0.688	0.691
São Paulo	0.658	0.663	0.669	0.675	0.68	0.686	0.69	0.694	0.698	0.702
Sergipe	0.532	0.539	0.546	0.553	0.56	0.566	0.57	0.575	0.579	0.583
Tocantins	0.514	0.524	0.534	0.544	0.552	0.56	0.567	0.573	0.579	0.583
Paraguay	0.582	0.588	0.594	0.601	0.608	0.615	0.621	0.627	0.633	0.638
North Africa and Middle East	0.595	0.603	0.611	0.619	0.626	0.633	0.64	0.647	0.654	0.66
North Africa and Middle East	0.595	0.603	0.611	0.619	0.626	0.633	0.64	0.647	0.654	0.66
Afghanistan	0.264	0.274	0.285	0.295	0.304	0.313	0.321	0.329	0.337	0.343
Algeria	0.599	0.605	0.611	0.617	0.623	0.628	0.634	0.64	0.646	0.652
Bahrain	0.711	0.715	0.72	0.726	0.731	0.735	0.739	0.743	0.747	0.751
Egypt	0.582	0.591	0.6	0.609	0.617	0.626	0.635	0.643	0.651	0.658
Iran	0.622	0.63	0.635	0.64	0.645	0.649	0.654	0.659	0.665	0.67
Iraq	0.57	0.583	0.597	0.61	0.622	0.632	0.644	0.654	0.663	0.671
Jordan	0.681	0.688	0.695	0.702	0.707	0.713	0.718	0.723	0.727	0.731
Kuwait	0.801	0.808	0.815	0.822	0.828	0.834	0.839	0.844	0.848	0.851
Lebanon	0.639	0.649	0.66	0.67	0.677	0.685	0.691	0.698	0.704	0.708
Libya	0.691	0.695	0.703	0.707	0.707	0.707	0.705	0.705	0.707	0.709
Morocco	0.475	0.483	0.491	0.499	0.508	0.516	0.524	0.533	0.541	0.548
Oman	0.715	0.726	0.737	0.747	0.754	0.76	0.767	0.773	0.778	0.783
Palestine	0.497	0.509	0.521	0.533	0.543	0.553	0.564	0.573	0.582	0.588
Qatar	0.772	0.779	0.786	0.793	0.799	0.806	0.812	0.818	0.825	0.83
Saudi Arabia	0.726	0.738	0.75	0.76	0.769	0.778	0.786	0.793	0.8	0.805
Sudan	0.416	0.428	0.44	0.451	0.462	0.474	0.485	0.497	0.507	0.515
Syria	0.594	0.601	0.605	0.606	0.607	0.608	0.61	0.613	0.616	0.619
Tunisia	0.622	0.628	0.634	0.64	0.646	0.651	0.657	0.662	0.667	0.672
Turkey	0.68	0.689	0.698	0.707	0.715	0.723	0.729	0.736	0.743	0.748
United Arab Emirates	0.853	0.859	0.863	0.868	0.87	0.872	0.874	0.876	0.879	0.88
Yemen	0.366	0.375	0.384	0.393	0.402	0.407	0.41	0.412	0.413	0.412
South Asia	0.456	0.465	0.475	0.485	0.495	0.505	0.515	0.525	0.535	0.543
South Asia	0.456	0.465	0.475	0.485	0.495	0.505	0.515	0.525	0.535	0.543
Bangladesh	0.408	0.416	0.425	0.433	0.441	0.449	0.457	0.466	0.475	0.483

Bhutan	0.384	0.394	0.403	0.411	0.419	0.426	0.434	0.442	0.449	0.455
India	0.473	0.483	0.493	0.504	0.515	0.526	0.537	0.547	0.558	0.566
Andhra Pradesh	0.451	0.461	0.471	0.481	0.492	0.504	0.515	0.527	0.537	0.546
Arunachal Pradesh	0.469	0.481	0.492	0.503	0.515	0.526	0.537	0.548	0.558	0.566
Assam	0.467	0.476	0.484	0.493	0.502	0.512	0.522	0.532	0.542	0.551
Bihar	0.346	0.356	0.367	0.378	0.389	0.4	0.412	0.423	0.434	0.444
Chhattisgarh	0.436	0.447	0.458	0.469	0.48	0.492	0.503	0.514	0.524	0.533
Delhi	0.634	0.643	0.652	0.661	0.671	0.682	0.692	0.701	0.71	0.717
Goa	0.655	0.663	0.669	0.676	0.682	0.689	0.697	0.704	0.711	0.717
Gujarat	0.515	0.524	0.534	0.545	0.557	0.569	0.58	0.591	0.601	0.609
Haryana	0.506	0.518	0.53	0.542	0.555	0.567	0.579	0.59	0.601	0.609
Himachal Pradesh	0.544	0.555	0.566	0.577	0.588	0.599	0.61	0.62	0.63	0.638
Jammu and Kashmir	0.518	0.529	0.539	0.549	0.558	0.568	0.578	0.587	0.596	0.605
Jharkhand	0.415	0.426	0.437	0.448	0.46	0.47	0.481	0.491	0.501	0.51
Karnataka	0.496	0.506	0.518	0.529	0.541	0.554	0.566	0.578	0.589	0.598
Kerala	0.587	0.595	0.604	0.614	0.625	0.636	0.646	0.655	0.664	0.671
Madhya Pradesh	0.405	0.415	0.425	0.436	0.448	0.46	0.472	0.484	0.495	0.505
Maharashtra	0.536	0.545	0.555	0.566	0.576	0.587	0.599	0.609	0.619	0.628
Manipur	0.527	0.534	0.541	0.548	0.556	0.564	0.572	0.58	0.588	0.595
Meghalaya	0.488	0.497	0.506	0.515	0.523	0.532	0.54	0.549	0.557	0.564
Mizoram	0.535	0.543	0.552	0.561	0.571	0.582	0.592	0.603	0.612	0.621
Nagaland	0.544	0.551	0.559	0.567	0.576	0.584	0.593	0.602	0.61	0.618
Odisha	0.452	0.462	0.472	0.482	0.493	0.504	0.514	0.524	0.534	0.542
Punjab	0.544	0.553	0.561	0.57	0.58	0.589	0.598	0.607	0.615	0.623
Rajasthan	0.415	0.427	0.439	0.451	0.464	0.476	0.489	0.501	0.512	0.521
Sikkim	0.535	0.55	0.564	0.577	0.589	0.601	0.612	0.622	0.632	0.64
Tamil Nadu	0.528	0.538	0.548	0.558	0.57	0.582	0.593	0.603	0.613	0.621
Telangana	0.467	0.479	0.491	0.503	0.516	0.528	0.54	0.552	0.563	0.572
Tripura	0.486	0.493	0.499	0.507	0.515	0.523	0.532	0.541	0.549	0.557
Union Territories other than Delhi	0.593	0.601	0.609	0.617	0.625	0.633	0.641	0.649	0.657	0.664
Uttar Pradesh	0.417	0.428	0.439	0.45	0.461	0.472	0.483	0.494	0.505	0.513
Uttarakhand	0.516	0.531	0.545	0.559	0.572	0.585	0.597	0.608	0.619	0.628
West Bengal	0.469	0.476	0.484	0.491	0.5	0.509	0.518	0.528	0.537	0.545
Nepal	0.347	0.356	0.365	0.373	0.382	0.391	0.399	0.408	0.416	0.422
Pakistan	0.379	0.387	0.394	0.402	0.41	0.418	0.426	0.434	0.442	0.449
Southeast Asia, east Asia, and Oceania	0.614	0.622	0.629	0.636	0.643	0.647	0.651	0.659	0.667	0.673
East Asia	0.628	0.637	0.644	0.652	0.66	0.662	0.665	0.675	0.684	0.691
China	0.621	0.631	0.638	0.646	0.654	0.657	0.659	0.669	0.679	0.686
North Korea	0.513	0.518	0.523	0.528	0.534	0.538	0.543	0.548	0.553	0.558
Taiwan (province of China)	0.83	0.833	0.838	0.843	0.848	0.852	0.856	0.86	0.865	0.868
Oceania	0.426	0.428	0.431	0.433	0.436	0.44	0.443	0.446	0.449	0.452
American Samoa	0.678	0.682	0.686	0.69	0.694	0.698	0.702	0.706	0.709	0.712
Cook Islands	0.722	0.726	0.73	0.734	0.739	0.744	0.75	0.755	0.76	0.764
Fiji	0.622	0.626	0.63	0.635	0.639	0.644	0.649	0.654	0.659	0.664
Guam	0.785	0.789	0.792	0.795	0.797	0.799	0.803	0.807	0.81	0.813

Kiribati	0.492	0.495	0.497	0.501	0.504	0.509	0.514	0.518	0.523	0.527
Marshall Islands	0.498	0.504	0.509	0.515	0.52	0.525	0.531	0.536	0.541	0.544
Federated States of Micronesia	0.546	0.55	0.554	0.558	0.561	0.565	0.569	0.573	0.577	0.58
Nauru	0.529	0.538	0.547	0.559	0.573	0.585	0.595	0.605	0.613	0.618
Niue	0.669	0.675	0.681	0.685	0.69	0.695	0.699	0.703	0.707	0.711
Northern Mariana Islands	0.751	0.75	0.75	0.75	0.75	0.751	0.756	0.761	0.767	0.771
Palau	0.707	0.71	0.714	0.717	0.72	0.725	0.729	0.732	0.735	0.738
Papua New Guinea	0.356	0.36	0.363	0.367	0.372	0.377	0.382	0.386	0.391	0.394
Samoa	0.612	0.615	0.618	0.62	0.623	0.626	0.629	0.633	0.637	0.641
Solomon Islands	0.358	0.364	0.371	0.377	0.382	0.387	0.393	0.398	0.403	0.407
Tokelau	0.565	0.573	0.58	0.588	0.595	0.602	0.608	0.615	0.621	0.626
Tonga	0.598	0.602	0.606	0.61	0.614	0.618	0.622	0.627	0.632	0.636
Tuvalu	0.545	0.549	0.553	0.558	0.562	0.567	0.573	0.579	0.584	0.589
Vanuatu	0.446	0.451	0.455	0.46	0.464	0.468	0.473	0.477	0.481	0.485
Southeast Asia	0.585	0.592	0.599	0.606	0.612	0.619	0.626	0.632	0.639	0.644
Cambodia	0.406	0.413	0.421	0.428	0.435	0.442	0.449	0.456	0.463	0.469
Indonesia	0.593	0.601	0.609	0.617	0.625	0.633	0.64	0.647	0.654	0.66
Aceh	0.608	0.614	0.62	0.626	0.633	0.64	0.646	0.653	0.66	0.666
Bali	0.582	0.59	0.598	0.606	0.613	0.621	0.628	0.635	0.642	0.648
Bangka-Belitung Islands	0.581	0.589	0.597	0.604	0.612	0.619	0.627	0.634	0.641	0.647
Banten	0.575	0.583	0.591	0.599	0.606	0.614	0.621	0.629	0.636	0.642
Bengkulu	0.55	0.558	0.567	0.575	0.583	0.591	0.598	0.606	0.613	0.619
Gorontalo	0.496	0.505	0.514	0.523	0.532	0.54	0.548	0.555	0.563	0.569
Jakarta	0.74	0.748	0.756	0.764	0.771	0.778	0.785	0.791	0.797	0.802
Jambi	0.571	0.581	0.59	0.598	0.606	0.614	0.621	0.628	0.635	0.641
West Java	0.582	0.59	0.598	0.606	0.614	0.621	0.629	0.636	0.643	0.648
Central Java	0.547	0.555	0.563	0.571	0.579	0.586	0.593	0.6	0.607	0.613
East Java	0.58	0.589	0.597	0.604	0.612	0.619	0.626	0.633	0.64	0.646
West Kalimantan	0.532	0.54	0.548	0.555	0.563	0.57	0.578	0.585	0.592	0.598
South Kalimantan	0.571	0.579	0.586	0.594	0.601	0.609	0.616	0.623	0.63	0.636
Central Kalimantan	0.589	0.596	0.604	0.611	0.619	0.626	0.633	0.64	0.647	0.653
East Kalimantan	0.695	0.703	0.711	0.719	0.727	0.734	0.742	0.749	0.756	0.762
North Kalimantan	0.689	0.698	0.707	0.715	0.723	0.731	0.738	0.745	0.752	0.758
Riau Islands	0.68	0.687	0.695	0.702	0.709	0.717	0.724	0.731	0.737	0.742
Lampung	0.546	0.556	0.565	0.574	0.582	0.59	0.598	0.605	0.612	0.617
Maluku	0.503	0.511	0.519	0.527	0.535	0.544	0.552	0.56	0.568	0.575
North Maluku	0.487	0.495	0.504	0.513	0.522	0.531	0.539	0.547	0.555	0.562
West Nusa Tenggara	0.513	0.522	0.53	0.538	0.546	0.554	0.561	0.568	0.575	0.582
East Nusa Tenggara	0.472	0.48	0.488	0.497	0.505	0.513	0.521	0.529	0.537	0.543
Papua	0.559	0.567	0.575	0.583	0.591	0.599	0.607	0.615	0.623	0.629
West Papua	0.58	0.593	0.604	0.615	0.625	0.634	0.643	0.652	0.66	0.666
Riau	0.663	0.67	0.678	0.685	0.693	0.7	0.707	0.714	0.721	0.727
West Sulawesi	0.495	0.505	0.515	0.524	0.533	0.542	0.55	0.558	0.566	0.573
South Sulawesi	0.554	0.564	0.573	0.581	0.59	0.598	0.605	0.613	0.62	0.626
Central Sulawesi	0.557	0.566	0.575	0.583	0.592	0.6	0.608	0.615	0.622	0.628
Southeast Sulawesi	0.536	0.546	0.556	0.565	0.574	0.582	0.59	0.598	0.606	0.612

North Sulawesi	0.6	0.608	0.615	0.623	0.631	0.638	0.645	0.652	0.658	0.664
West Sumatra	0.6	0.609	0.617	0.625	0.633	0.641	0.648	0.655	0.662	0.668
South Sumatra	0.59	0.598	0.607	0.615	0.622	0.63	0.637	0.644	0.651	0.657
North Sumatra	0.605	0.614	0.623	0.631	0.639	0.647	0.654	0.662	0.669	0.675
Yogyakarta	0.608	0.616	0.624	0.631	0.639	0.646	0.652	0.659	0.665	0.671
Laos	0.413	0.422	0.431	0.441	0.45	0.458	0.467	0.475	0.483	0.49
Malaysia	0.693	0.698	0.704	0.71	0.716	0.722	0.726	0.728	0.732	0.737
Maldives	0.504	0.511	0.518	0.525	0.532	0.538	0.544	0.551	0.557	0.562
Mauritius	0.652	0.658	0.665	0.673	0.68	0.686	0.69	0.695	0.7	0.705
Myanmar	0.446	0.455	0.464	0.473	0.482	0.49	0.498	0.506	0.514	0.521
Philippines	0.567	0.572	0.577	0.583	0.589	0.596	0.603	0.61	0.617	0.623
Seychelles	0.679	0.683	0.687	0.691	0.696	0.702	0.707	0.713	0.719	0.724
Sri Lanka	0.628	0.636	0.644	0.651	0.658	0.666	0.672	0.678	0.684	0.69
Thailand	0.638	0.643	0.649	0.655	0.66	0.666	0.671	0.676	0.682	0.687
Timor-Leste	0.458	0.469	0.48	0.488	0.493	0.498	0.503	0.508	0.511	0.514
Vietnam	0.549	0.558	0.566	0.573	0.581	0.589	0.596	0.604	0.611	0.617
Sub-Saharan Africa	0.394	0.401	0.409	0.416	0.423	0.431	0.438	0.445	0.452	0.456
Central sub-Saharan Africa	0.378	0.389	0.4	0.412	0.423	0.434	0.445	0.454	0.463	0.47
Angola	0.376	0.387	0.398	0.41	0.421	0.432	0.443	0.454	0.463	0.47
Central African Republic	0.253	0.258	0.263	0.263	0.263	0.264	0.266	0.268	0.271	0.274
Congo (Brazzaville)	0.491	0.5	0.509	0.519	0.528	0.538	0.547	0.556	0.563	0.568
DR Congo	0.266	0.277	0.289	0.305	0.321	0.336	0.35	0.362	0.374	0.382
Equatorial Guinea	0.578	0.594	0.611	0.626	0.64	0.652	0.663	0.673	0.681	0.685
Gabon	0.579	0.587	0.596	0.605	0.614	0.623	0.632	0.641	0.649	0.656
Eastern sub-Saharan Africa	0.336	0.343	0.351	0.359	0.367	0.375	0.383	0.391	0.399	0.405
Burundi	0.243	0.248	0.254	0.26	0.266	0.27	0.274	0.278	0.282	0.284
Comoros	0.401	0.407	0.413	0.419	0.426	0.432	0.438	0.444	0.45	0.455
Djibouti	0.384	0.392	0.4	0.408	0.416	0.425	0.434	0.443	0.452	0.459
Eritrea	0.334	0.34	0.346	0.353	0.36	0.367	0.375	0.382	0.39	0.396
Ethiopia	0.244	0.256	0.268	0.279	0.291	0.302	0.313	0.324	0.334	0.343
Kenya	0.441	0.448	0.455	0.463	0.47	0.478	0.486	0.494	0.502	0.508
Baringo	0.392	0.4	0.408	0.417	0.426	0.436	0.447	0.457	0.468	0.476
Bomet	0.433	0.443	0.454	0.465	0.476	0.487	0.499	0.511	0.522	0.531
Bungoma	0.395	0.404	0.413	0.423	0.433	0.444	0.454	0.465	0.475	0.483
Busia	0.37	0.378	0.387	0.396	0.405	0.415	0.425	0.435	0.445	0.453
Elgeyo Marakwet	0.406	0.416	0.426	0.437	0.449	0.46	0.471	0.483	0.494	0.503
Embu	0.478	0.485	0.492	0.5	0.508	0.516	0.525	0.534	0.542	0.55
Garissa	0.259	0.264	0.27	0.275	0.281	0.287	0.293	0.299	0.305	0.31
Homa Bay	0.348	0.358	0.369	0.381	0.393	0.405	0.418	0.431	0.443	0.452
Isiolo	0.339	0.346	0.353	0.361	0.369	0.378	0.387	0.396	0.405	0.413
Kajiado	0.48	0.489	0.498	0.507	0.516	0.526	0.536	0.545	0.555	0.563
Kakamega	0.401	0.41	0.419	0.429	0.439	0.449	0.46	0.47	0.481	0.489
Kericho	0.408	0.419	0.43	0.441	0.453	0.465	0.477	0.489	0.501	0.509
Kiambu	0.538	0.546	0.554	0.562	0.57	0.578	0.586	0.594	0.602	0.609
Kilifi	0.407	0.415	0.424	0.433	0.443	0.453	0.463	0.474	0.484	0.492
Kirinyaga	0.477	0.484	0.491	0.499	0.506	0.514	0.523	0.531	0.539	0.546

Kisii	0.468	0.477	0.487	0.497	0.507	0.518	0.528	0.539	0.549	0.557
Kisumu	0.465	0.475	0.485	0.496	0.507	0.518	0.529	0.54	0.551	0.559
Kitui	0.388	0.396	0.406	0.415	0.425	0.435	0.446	0.456	0.466	0.474
Kwale	0.39	0.397	0.405	0.414	0.423	0.432	0.442	0.452	0.461	0.469
Laikipia	0.479	0.489	0.498	0.508	0.518	0.528	0.538	0.549	0.559	0.567
Lamu	0.414	0.421	0.429	0.436	0.444	0.453	0.462	0.471	0.48	0.488
Machakos	0.472	0.48	0.489	0.498	0.508	0.517	0.527	0.536	0.546	0.553
Makueni	0.381	0.389	0.398	0.406	0.416	0.425	0.435	0.445	0.455	0.462
Mandera	0.245	0.251	0.257	0.264	0.27	0.277	0.283	0.29	0.296	0.302
Marsabit	0.313	0.32	0.328	0.337	0.346	0.354	0.363	0.372	0.381	0.388
Meru	0.448	0.456	0.464	0.472	0.481	0.49	0.5	0.509	0.518	0.525
Migori	0.345	0.354	0.364	0.375	0.386	0.397	0.409	0.421	0.433	0.442
Mombasa	0.507	0.514	0.522	0.53	0.539	0.548	0.557	0.566	0.575	0.582
Murang'a	0.478	0.486	0.493	0.501	0.509	0.518	0.526	0.535	0.543	0.55
Nairobi	0.604	0.61	0.617	0.624	0.63	0.637	0.645	0.652	0.66	0.665
Nakuru	0.461	0.47	0.479	0.489	0.499	0.509	0.52	0.53	0.54	0.548
Nandi	0.453	0.462	0.471	0.481	0.491	0.502	0.513	0.524	0.534	0.543
Narok	0.33	0.338	0.347	0.356	0.366	0.376	0.387	0.398	0.409	0.418
Nyamira	0.487	0.496	0.506	0.517	0.527	0.538	0.55	0.561	0.571	0.579
Nyandarua	0.467	0.475	0.483	0.492	0.501	0.51	0.519	0.529	0.538	0.546
Nyeri	0.496	0.504	0.512	0.52	0.528	0.536	0.545	0.553	0.561	0.568
Samburu	0.281	0.289	0.297	0.306	0.314	0.324	0.333	0.343	0.353	0.361
Siaya	0.347	0.358	0.368	0.38	0.392	0.404	0.416	0.428	0.44	0.449
Taita Taveta	0.462	0.47	0.478	0.486	0.495	0.504	0.513	0.522	0.531	0.539
Tana River	0.317	0.324	0.331	0.34	0.348	0.357	0.366	0.375	0.384	0.391
Tharaka Nithi	0.479	0.488	0.497	0.506	0.516	0.526	0.536	0.546	0.556	0.564
Trans Nzoia	0.451	0.459	0.468	0.478	0.488	0.498	0.509	0.52	0.53	0.539
Turkana	0.286	0.292	0.299	0.306	0.314	0.322	0.33	0.339	0.347	0.355
Uasin Gishu	0.496	0.505	0.514	0.524	0.535	0.545	0.556	0.566	0.577	0.585
Vihiga	0.391	0.398	0.405	0.413	0.421	0.429	0.438	0.447	0.457	0.464
Wajir	0.21	0.215	0.22	0.225	0.231	0.236	0.242	0.248	0.253	0.259
West Pokot	0.326	0.336	0.346	0.357	0.368	0.379	0.39	0.402	0.413	0.422
Madagascar	0.336	0.342	0.348	0.355	0.361	0.369	0.376	0.383	0.391	0.396
Malawi	0.317	0.326	0.335	0.342	0.35	0.358	0.365	0.372	0.379	0.384
Mozambique	0.237	0.244	0.252	0.26	0.268	0.277	0.285	0.294	0.301	0.307
Rwanda	0.359	0.368	0.376	0.384	0.391	0.399	0.407	0.415	0.422	0.429
Somalia	0.0692	0.0703	0.0716	0.0728	0.0742	0.0756	0.077	0.0785	0.0799	0.081
South Sudan	0.32	0.325	0.33	0.334	0.339	0.342	0.345	0.351	0.358	0.363
Uganda	0.325	0.335	0.345	0.355	0.364	0.373	0.382	0.391	0.399	0.404
Tanzania	0.354	0.361	0.368	0.375	0.383	0.391	0.399	0.408	0.416	0.423
Zambia	0.418	0.429	0.44	0.451	0.462	0.472	0.481	0.491	0.499	0.505
Southern sub-Saharan Africa	0.605	0.61	0.614	0.619	0.623	0.628	0.632	0.636	0.639	0.642
Botswana	0.581	0.587	0.593	0.6	0.606	0.612	0.618	0.624	0.63	0.634
eSwatini	0.526	0.532	0.538	0.545	0.551	0.557	0.563	0.569	0.574	0.577
Lesotho	0.448	0.455	0.462	0.469	0.476	0.483	0.489	0.496	0.502	0.507
Namibia	0.558	0.564	0.571	0.577	0.584	0.591	0.597	0.603	0.608	0.612
South Africa	0.642	0.647	0.651	0.656	0.66	0.664	0.668	0.672	0.676	0.678

Zimbabwe	0.425	0.427	0.432	0.438	0.445	0.452	0.459	0.465	0.471	0.476
Western sub-Saharan Africa	0.383	0.391	0.399	0.407	0.415	0.422	0.43	0.437	0.443	0.448
Benin	0.297	0.301	0.306	0.312	0.318	0.324	0.331	0.338	0.346	0.352
Burkina Faso	0.21	0.215	0.22	0.226	0.231	0.236	0.241	0.247	0.252	0.257
Cape Verde	0.461	0.469	0.477	0.484	0.491	0.498	0.505	0.512	0.519	0.525
Cameroon	0.412	0.42	0.428	0.436	0.445	0.455	0.464	0.474	0.483	0.49
Chad	0.185	0.191	0.197	0.203	0.21	0.216	0.223	0.228	0.234	0.238
Côte d'Ivoire	0.346	0.35	0.355	0.362	0.369	0.376	0.384	0.393	0.401	0.408
The Gambia	0.334	0.341	0.348	0.356	0.363	0.37	0.378	0.385	0.393	0.399
Ghana	0.474	0.484	0.494	0.504	0.514	0.523	0.531	0.541	0.549	0.557
Guinea	0.267	0.272	0.278	0.284	0.29	0.296	0.303	0.31	0.318	0.325
Guinea-Bissau	0.297	0.304	0.31	0.316	0.322	0.328	0.335	0.342	0.349	0.355
Liberia	0.296	0.305	0.314	0.325	0.335	0.344	0.351	0.358	0.365	0.37
Mali	0.214	0.22	0.225	0.23	0.235	0.241	0.247	0.253	0.259	0.263
Mauritania	0.427	0.435	0.443	0.45	0.459	0.467	0.474	0.482	0.49	0.496
Niger	0.119	0.123	0.128	0.133	0.138	0.143	0.148	0.153	0.158	0.162
Nigeria	0.442	0.451	0.46	0.469	0.478	0.487	0.495	0.503	0.51	0.515
São Tomé and PrÍncipe	0.424	0.433	0.443	0.452	0.461	0.47	0.478	0.487	0.495	0.502
Senegal	0.33	0.336	0.342	0.348	0.354	0.361	0.368	0.375	0.382	0.389
Sierra Leone	0.275	0.283	0.292	0.304	0.314	0.321	0.328	0.335	0.342	0.347
Togo	0.352	0.358	0.364	0.371	0.379	0.386	0.394	0.402	0.411	0.417

Data obtained from Global Burden of Disease Collaborative Network. Global Burden of Disease Study 2019 (GBD 2019) Socio-Demographic Index (SDI) 1950–2019. Seattle, United States of America: Institute for Health Metrics and Evaluation (IHME), 2020.

Supplementary Material Table S2 Data quality rating for cause of death data 2010-2019, by country.

Country	Data Quality Rating
Afghanistan	1
Albania	3
Algeria	1
American Samoa	3
Andorra	1
Angola	1
Antigua and Barbuda	4
Argentina	4
Armenia	5
Australia	5
Austria	5
Azerbaijan	3
Bahrain	3
Bangladesh	2
Barbados	4
Belarus	4
Belgium	4
Belize	4
Benin	1
Bermuda	5
Bhutan	0
Venezuela	5
Bosnia and Herzegovina	2
Botswana	0
Brazil	4
Brunei	3
Bulgaria	4
Burkina Faso	1
Burundi	1
Cambodia	1
Cameroon	0
Canada	5
Central African Republic	0
Chad	0
Chile	4
China	3
Colombia	4
The Bahamas	4
Comoros	0
Congo (Brazzaville)	0
Cook Islands	2
Costa Rica	5

Croatia	4
Cuba	5
Cyprus	2
Czech Republic	4
North Korea	0
DR Congo	1
Denmark	5
Djibouti	0
Dominica	3
Dominican Republic	3
Ecuador	3
Egypt	2
El Salvador	3
Equatorial Guinea	0
Eritrea	0
Estonia	5
Ethiopia	1
Federated States of Micronesia	0
Fiji	2
Finland	5
France	4
Gabon	0
Georgia	4
Germany	4
Ghana	1
Greece	4
Greenland	3
Grenada	4
Guam	3
Guatemala	4
Guinea	1
Guinea-Bissau	1
Guyana	4
Haiti	1
Honduras	2
Hungary	5
Iceland	5
India	2
Indonesia	2
Iraq	2
Ireland	5
Iran	2
Israel	4
Italy	5
Jamaica	4

Japan	5
Jordan	2
Kazakhstan	4
Kenya	1
eSwatini	1
Kiribati	2
Kuwait	4
Kyrgyzstan	4
Laos	1
Latvia	5
Lebanon	1
Lesotho	0
Liberia	1
Libya	1
Lithuania	5
Luxembourg	4
Madagascar	1
Malawi	1
Malaysia	2
Maldives	2
Mali	1
Malta	5
Marshall Islands	0
Mauritania	0
Mauritius	4
Mexico	4
Mongolia	2
Montenegro	2
Morocco	2
Mozambique	2
Myanmar	1
Namibia	0
Nepal	1
Netherlands	5
New Zealand	5
Nicaragua	3
Niger	1
Nigeria	1
Northern Mariana Islands	2
Norway	5
Oman	2
Pakistan	2
Palestine	2
Panama	4
Papua New Guinea	1

Paraguay	3
Peru	3
Philippines	3
Bolivia	1
Poland	4
Portugal	4
Monaco	2
Puerto Rico	4
Qatar	2
Cape Verde	2
Côte d'Ivoire	1
South Korea	3
Moldova	5
Nauru	0
Niue	0
Palau	1
San Marino	3
The Gambia	1
Romania	4
Russia	5
Rwanda	1
Saint Kitts and Nevis	4
Saint Lucia	4
Saint Vincent and the Grenadines	4
Samoa	0
São Tomé and PrÍncipe	1
Saudi Arabia	2
Senegal	1
Serbia	3
Seychelles	3
Sierra Leone	1
Singapore	5
Slovakia	3
Slovenia	4
Vietnam	2
Solomon Islands	1
Somalia	0
South Africa	3
South Sudan	0
Spain	4
Sri Lanka	3
Sudan	0
Suriname	3
Sweden	5

Switzerland	4
Syria	3
Taiwan (province of China)	4
Tajikistan	3
Thailand	3
North Macedonia	3
Timor-Leste	0
Togo	0
Tokelau	0
Tonga	1
Trinidad and Tobago	5
Tunisia	1
Turkey	3
Turkmenistan	4
Tuvalu	0
Uganda	1
Ukraine	5
United Arab Emirates	1
UK	5
Northern Ireland	5
Scotland	5
Wales	5
England	5
Tanzania	1
Virgin Islands	3
USA	5
Uruguay	4
Uzbekistan	4
Vanuatu	0
Yemen	0
Zambia	1
Zimbabwe	2

Data obtained from Global Burden of Disease Collaborative Network. Global Burden of Disease Study 2019 (GBD 2019). Seattle, United States of America: Institute for Health Metrics and Evaluation (IHME), 2020.

Supplementary Material Table S3. Age-specific incidence rates (ASIRs) of inflammatory bowel disease in patient aged 65-89 years in 2010 and 2019, stratified by countries.

Countries	2010 ASIR, per 100,000 (95% UI)	2019 ASIR, per 100,000 (95% UI)	APC (95% CI)	***p***
Afghanistan	2.67 (1.87 to 3.8)	2.87 (2.05 to 4.07)	0.78 (0.65 to 0.9)	<0.001
Albania	10.68 (8.07 to 14.32)	10.54 (7.86 to 13.74)	-0.15 (-0.19 to -0.12)	<0.001
Algeria	5.15 (3.66 to 7.41)	5.44 (3.78 to 7.47)	0.59 (0.52 to 0.65)	<0.001
American Samoa	0.91 (0.68 to 1.23)	0.99 (0.74 to 1.32)	0.79 (0.34 to 1.24)	0.001
Andorra	10.06 (7.75 to 13.04)	9.9 (7.68 to 12.79)	-0.15 (-0.28 to -0.03)	0.017
Angola	2.27 (1.69 to 3)	2.42 (1.81 to 3.19)	0.71 (0.64 to 0.77)	<0.001
Antigua and Barbuda	3.09 (2.23 to 4.25)	3.69 (2.65 to 5.11)	2.03 (1.94 to 2.12)	<0.001
Argentina	1.61 (1.23 to 2.09)	1.67 (1.25 to 2.19)	0.36 (0.18 to 0.55)	<0.001
Armenia	10.02 (7.57 to 13.46)	10.46 (7.86 to 14.03)	0.44 (0.34 to 0.54)	<0.001
Australia	18.36 (15.28 to 21.62)	20.02 (16.68 to 23.72)	1.02 (0.84 to 1.2)	<0.001
Austria	12.77 (9.83 to 17.05)	12.09 (9.56 to 15.53)	-0.56 (-0.66 to -0.45)	<0.001
Azerbaijan	10.84 (8.17 to 14.4)	11.03 (8.22 to 14.63)	0.19 (-0.01 to 0.38)	0.061
Bahamas	3.76 (2.78 to 5.29)	4.71 (3.36 to 6.5)	2.68 (2.56 to 2.8)	<0.001
Bahrain	4.28 (3.06 to 6)	4.52 (3.18 to 6.36)	0.58 (0.51 to 0.66)	<0.001
Bangladesh	2.95 (2.12 to 4.01)	3 (2.2 to 4.12)	0.15 (0.03 to 0.27)	0.017
Barbados	4.27 (3 to 6.11)	5.16 (3.6 to 7.13)	2.11 (1.96 to 2.25)	<0.001
Belarus	6.45 (4.73 to 8.9)	7.32 (5.39 to 9.77)	1.53 (1.26 to 1.8)	<0.001
Belgium	7.82 (6.66 to 9.19)	10.7 (8.28 to 13.83)	3.71 (3.15 to 4.28)	<0.001
Belize	3.28 (2.44 to 4.54)	3.88 (2.83 to 5.37)	1.97 (1.87 to 2.08)	<0.001
Benin	1.93 (1.42 to 2.6)	2.15 (1.59 to 2.83)	1.2 (0.97 to 1.42)	<0.001
Bermuda	3.86 (2.77 to 5.29)	4.46 (3.26 to 6.07)	1.57 (1.32 to 1.81)	<0.001
Bhutan	2.15 (1.59 to 3)	2.44 (1.74 to 3.49)	1.45 (1.25 to 1.65)	<0.001
Bolivia (Plurinational State of)	3.46 (2.59 to 4.49)	3.85 (2.91 to 4.98)	1.21 (0.99 to 1.44)	<0.001
Bosnia and Herzegovina	11.33 (8.84 to 14.47)	11.78 (9.17 to 14.87)	0.45 (0.4 to 0.5)	<0.001
Botswana	2.52 (1.82 to 3.57)	2.65 (1.97 to 3.56)	0.54 (0.44 to 0.64)	<0.001
Brazil	10.91 (8.9 to 13.48)	10.67 (8.58 to 13.28)	-0.07 (-0.35 to 0.21)	0.592
Brunei Darussalam	3.8 (2.66 to 5.24)	3.98 (2.83 to 5.56)	0.52 (0.46 to 0.58)	<0.001
Bulgaria	11.38 (8.65 to 14.95)	11.53 (8.65 to 15.11)	0.17 (0.13 to 0.2)	<0.001
Burkina Faso	1.76 (1.31 to 2.37)	1.9 (1.39 to 2.53)	0.87 (0.79 to 0.95)	<0.001
Burundi	1.69 (1.24 to 2.28)	1.69 (1.26 to 2.28)	0.02 (0.01 to 0.04)	0.015
C?¥te d'Ivoire	2.05 (1.5 to 2.78)	2.3 (1.7 to 3.09)	1.38 (1.16 to 1.6)	<0.001
Cabo Verde	1.92 (1.39 to 2.6)	2.19 (1.66 to 2.97)	1.51 (1.29 to 1.72)	<0.001
Cambodia	0.53 (0.38 to 0.7)	0.57 (0.42 to 0.76)	0.83 (0.57 to 1.08)	<0.001
Cameroon	2.21 (1.64 to 2.95)	2.35 (1.72 to 3.23)	0.63 (0.36 to 0.9)	<0.001
Canada	36.84 (35.2 to 38.54)	33.34 (30.99 to 35.94)	-1.13 (-1.36 to -0.9)	<0.001

Central African Republic	1.94 (1.45 to 2.63)	2 (1.5 to 2.69)	0.37 (0.32 to 0.41)	<0.001
Chad	1.53 (1.12 to 2.14)	1.79 (1.31 to 2.5)	1.76 (1.42 to 2.1)	<0.001
Chile	1.73 (1.29 to 2.29)	1.75 (1.31 to 2.34)	0.14 (-0.1 to 0.38)	0.248
China	2.36 (1.75 to 3.19)	3.02 (2.23 to 4.11)	2.72 (2.54 to 2.89)	<0.001
Colombia	2.83 (2.15 to 3.8)	3.24 (2.45 to 4.3)	1.53 (1.26 to 1.8)	<0.001
Comoros	1.85 (1.39 to 2.43)	2.01 (1.51 to 2.67)	0.99 (0.89 to 1.09)	<0.001
Congo	2.36 (1.75 to 3.23)	2.54 (1.84 to 3.37)	0.88 (0.77 to 0.99)	<0.001
Cook Islands	1.06 (0.79 to 1.43)	1.19 (0.9 to 1.57)	1.19 (1.12 to 1.27)	<0.001
Costa Rica	3.46 (2.61 to 4.61)	3.71 (2.83 to 4.84)	0.71 (0.58 to 0.85)	<0.001
Croatia	10.01 (8.42 to 11.75)	11.16 (8.97 to 14.17)	1.24 (1.2 to 1.28)	<0.001
Cuba	3.07 (2.25 to 4.22)	3.65 (2.67 to 5.06)	2.04 (1.92 to 2.16)	<0.001
Cyprus	5.28 (4.42 to 6.24)	5.34 (4.51 to 6.38)	0.12 (0.09 to 0.15)	<0.001
Czechia	6.55 (5.55 to 7.63)	7.94 (6.82 to 9.31)	2.48 (2.1 to 2.85)	<0.001
Democratic People's Republic of Korea	1.31 (0.94 to 1.83)	1.4 (1.02 to 1.88)	0.76 (0.73 to 0.8)	<0.001
Democratic Republic of the Congo	1.94 (1.43 to 2.58)	2.21 (1.65 to 2.93)	1.55 (1.29 to 1.8)	<0.001
Denmark	20.4 (18.07 to 22.71)	19.48 (16.7 to 22.34)	-0.47 (-0.55 to -0.38)	<0.001
Djibouti	1.81 (1.37 to 2.4)	1.75 (1.3 to 2.34)	-0.33 (-0.44 to -0.22)	<0.001
Dominica	4.08 (3.04 to 5.55)	4.46 (3.2 to 6.13)	0.98 (0.85 to 1.12)	<0.001
Dominican Republic	3.2 (2.28 to 4.39)	3.79 (2.77 to 5.15)	1.95 (1.78 to 2.12)	<0.001
Ecuador	2.94 (2.27 to 3.78)	3.08 (2.39 to 3.91)	0.42 (0.28 to 0.56)	<0.001
Egypt	5.66 (4.02 to 7.79)	6.26 (4.54 to 8.49)	1.16 (1.08 to 1.23)	<0.001
El Salvador	3.65 (2.77 to 4.75)	3.68 (2.82 to 4.76)	0.14 (0 to 0.28)	0.048
Equatorial Guinea	2.6 (1.93 to 3.47)	2.95 (2.25 to 3.91)	1.39 (1.27 to 1.5)	<0.001
Eritrea	1.81 (1.36 to 2.45)	1.89 (1.42 to 2.53)	0.5 (0.43 to 0.58)	<0.001
Estonia	6.34 (5.11 to 7.88)	7.97 (6.36 to 9.86)	2.7 (2.4 to 3)	<0.001
Eswatini	2.56 (1.89 to 3.45)	2.71 (1.99 to 3.65)	0.71 (0.58 to 0.84)	<0.001
Ethiopia	1.77 (1.34 to 2.36)	1.94 (1.46 to 2.56)	0.98 (0.93 to 1.02)	<0.001
Fiji	1.02 (0.76 to 1.35)	1.12 (0.84 to 1.47)	1.03 (0.99 to 1.08)	<0.001
Finland	14.97 (13.34 to 16.7)	23.57 (18.97 to 28.49)	5.11 (4.75 to 5.47)	<0.001
France	8.56 (7.95 to 9.18)	6.99 (6.29 to 7.74)	-1.92 (-2.6 to -1.24)	<0.001
Gabon	2.59 (1.89 to 3.45)	2.9 (2.13 to 3.87)	1.27 (1.2 to 1.34)	<0.001
Gambia	2.01 (1.43 to 2.75)	2.24 (1.69 to 3.01)	1.22 (1.16 to 1.27)	<0.001
Georgia	9.54 (7.16 to 12.98)	9.28 (7.04 to 12.41)	-0.31 (-0.48 to -0.13)	0.001
Germany	10.77 (8.34 to 14.17)	11.65 (9.12 to 15.14)	0.9 (0.83 to 0.97)	<0.001
Ghana	1.99 (1.45 to 2.81)	2.16 (1.59 to 2.97)	0.63 (0.2 to 1.05)	0.009
Greece	7.18 (6.12 to 8.35)	7.55 (6.52 to 8.72)	0.56 (0.47 to 0.64)	<0.001
Greenland	31.63 (24.48 to 39.47)	31.21 (24.28 to 39.2)	-0.17 (-0.21 to -0.13)	<0.001
Grenada	3.9 (2.87 to 5.39)	4.46 (3.37 to 6.19)	1.5 (1.46 to 1.55)	<0.001
Guam	1.08 (0.82 to 1.44)	1.2 (0.9 to 1.57)	1.11 (1.06 to 1.15)	<0.001
Guatemala	3.27 (2.45 to 4.31)	3.48 (2.63 to 4.6)	0.67 (0.58 to 0.75)	<0.001

Guinea	1.72 (1.26 to 2.35)	1.93 (1.4 to 2.55)	1.31 (1.1 to 1.52)	<0.001
Guinea-Bissau	1.81 (1.32 to 2.44)	2.02 (1.51 to 2.78)	1.24 (1.02 to 1.45)	<0.001
Guyana	3.36 (2.45 to 4.68)	4.1 (2.99 to 5.66)	2.22 (2.03 to 2.4)	<0.001
Haiti	2.72 (1.92 to 3.82)	3.28 (2.37 to 4.49)	2.11 (1.96 to 2.26)	<0.001
Honduras	3.07 (2.33 to 4.03)	3.28 (2.47 to 4.32)	0.74 (0.6 to 0.88)	<0.001
Hungary	14.63 (13.44 to 15.89)	18.79 (16.8 to 21.04)	3.04 (2.77 to 3.3)	<0.001
Iceland	16.47 (13.01 to 20.86)	17.06 (13.16 to 21.56)	0.34 (0.29 to 0.4)	<0.001
India	3.82 (2.8 to 5.21)	4.08 (3 to 5.47)	0.62 (0.2 to 1.04)	0.004
Indonesia	0.71 (0.54 to 0.94)	0.77 (0.58 to 1.03)	0.71 (0.59 to 0.83)	<0.001
Iran (Islamic Republic of)	5.75 (3.98 to 8.12)	5.58 (3.85 to 7.83)	-0.34 (-0.39 to -0.3)	<0.001
Iraq	2.64 (1.91 to 3.77)	3.05 (2.2 to 4.31)	1.46 (1.28 to 1.64)	<0.001
Ireland	8.59 (6.51 to 11.38)	9.28 (7.12 to 12.21)	0.93 (0.73 to 1.14)	<0.001
Israel	6.38 (5.37 to 7.52)	6.81 (5.73 to 8)	0.73 (0.69 to 0.78)	<0.001
Italy	17.15 (13.42 to 21.68)	16.32 (12.69 to 20.8)	-0.45 (-0.58 to -0.32)	<0.001
Jamaica	2.73 (1.97 to 3.8)	3.51 (2.52 to 4.87)	3.21 (2.85 to 3.57)	<0.001
Japan	7.02 (5.07 to 9.55)	6.93 (4.96 to 9.43)	-0.13 (-0.28 to 0.03)	0.115
Jordan	5.95 (4.09 to 8.41)	6.88 (4.73 to 10.33)	1.43 (1.1 to 1.76)	<0.001
Kazakhstan	8.98 (6.72 to 12.06)	10.85 (8.15 to 14.78)	2.25 (1.78 to 2.71)	<0.001
Kenya	1.78 (1.36 to 2.33)	2.32 (1.76 to 3.04)	2.81 (2.64 to 2.99)	<0.001
Kiribati	0.78 (0.57 to 1.07)	0.85 (0.63 to 1.13)	0.86 (0.79 to 0.92)	<0.001
Kuwait	4.6 (3.39 to 6.21)	5.11 (3.65 to 7.17)	1.18 (1.1 to 1.25)	<0.001
Kyrgyzstan	9.34 (7.05 to 12.4)	9.6 (7.19 to 12.98)	0.12 (-0.15 to 0.39)	0.335
Lao People's Democratic Republic	0.52 (0.38 to 0.71)	0.61 (0.45 to 0.81)	1.7 (1.64 to 1.76)	<0.001
Latvia	7.52 (5.64 to 9.99)	7.44 (5.58 to 9.82)	-0.11 (-0.17 to -0.04)	0.003
Lebanon	4.18 (2.88 to 6)	4.49 (3.17 to 6.27)	0.71 (0.62 to 0.8)	<0.001
Lesotho	2.29 (1.66 to 3.12)	2.44 (1.81 to 3.2)	0.75 (0.59 to 0.9)	<0.001
Liberia	1.91 (1.39 to 2.65)	2.18 (1.61 to 2.91)	1.49 (1.39 to 1.58)	<0.001
Libya	2.96 (2.08 to 4.13)	3.52 (2.51 to 4.88)	2.01 (1.56 to 2.46)	<0.001
Lithuania	7.58 (6.6 to 8.58)	8.34 (7.34 to 9.36)	1.13 (1.06 to 1.21)	<0.001
Luxembourg	14.83 (11.17 to 19.41)	14.95 (11.5 to 19.66)	0.2 (0.01 to 0.39)	0.039
Madagascar	1.82 (1.38 to 2.35)	1.89 (1.4 to 2.52)	0.48 (0.42 to 0.54)	<0.001
Malawi	1.74 (1.3 to 2.32)	1.89 (1.42 to 2.48)	0.94 (0.85 to 1.03)	<0.001
Malaysia	0.73 (0.6 to 0.88)	0.84 (0.68 to 1.04)	1.63 (1.58 to 1.68)	<0.001
Maldives	0.56 (0.41 to 0.76)	0.63 (0.46 to 0.84)	1.31 (1.22 to 1.4)	<0.001
Mali	1.67 (1.23 to 2.28)	1.89 (1.38 to 2.51)	1.39 (1.13 to 1.65)	<0.001
Malta	7.26 (5.78 to 9.03)	7.45 (5.94 to 9.27)	0.28 (0.23 to 0.33)	<0.001
Marshall Islands	0.87 (0.64 to 1.15)	0.97 (0.72 to 1.29)	1.25 (1.19 to 1.31)	<0.001
Mauritania	1.93 (1.4 to 2.62)	2.04 (1.5 to 2.81)	0.64 (0.48 to 0.81)	<0.001
Mauritius	0.66 (0.49 to 0.89)	0.74 (0.56 to 0.98)	1.23 (1.17 to 1.3)	<0.001
Mexico	4.66 (3.62 to 6.02)	5.1 (3.93 to 6.57)	0.99 (0.91 to 1.08)	<0.001

Micronesia (Federated States of)	0.91 (0.67 to 1.21)	1.01 (0.77 to 1.37)	1.26 (1.21 to 1.31)	<0.001
Monaco	10.16 (7.7 to 13.31)	10.19 (7.76 to 13.38)	-0.01 (-0.22 to 0.19)	0.901
Mongolia	10.76 (7.86 to 14.66)	11.19 (8.49 to 14.91)	0.3 (0.13 to 0.47)	0.004
Montenegro	10.09 (7.47 to 13.43)	10.45 (8.05 to 13.84)	0.38 (0.2 to 0.56)	<0.001
Morocco	1.99 (1.46 to 2.64)	2.15 (1.58 to 2.97)	0.86 (0.8 to 0.93)	<0.001
Mozambique	1.63 (1.23 to 2.14)	1.73 (1.28 to 2.29)	0.64 (0.52 to 0.75)	<0.001
Myanmar	0.56 (0.41 to 0.74)	0.65 (0.48 to 0.85)	1.7 (1.63 to 1.76)	<0.001
Namibia	2.59 (1.93 to 3.46)	2.49 (1.86 to 3.33)	-0.42 (-0.47 to -0.37)	<0.001
Nauru	0.98 (0.74 to 1.31)	0.92 (0.68 to 1.25)	-0.81 (-1.24 to -0.38)	<0.001
Nepal	2.84 (2.05 to 3.87)	3.13 (2.31 to 4.26)	1.23 (0.79 to 1.68)	<0.001
Netherlands	11.5 (9.71 to 13.47)	8.59 (6.74 to 10.82)	-3.29 (-3.87 to -2.71)	<0.001
New Zealand	21.55 (17.25 to 26.13)	21.64 (17.43 to 26.5)	0.12 (0.04 to 0.2)	0.008
Nicaragua	3.44 (2.63 to 4.41)	3.1 (2.33 to 4.06)	-1.24 (-1.45 to -1.03)	<0.001
Niger	1.46 (1.05 to 2.03)	1.62 (1.19 to 2.2)	1.22 (0.98 to 1.46)	<0.001
Nigeria	2.17 (1.61 to 2.96)	2.6 (1.93 to 3.47)	2.1 (1.75 to 2.46)	<0.001
Niue	1.01 (0.77 to 1.33)	1.11 (0.84 to 1.46)	1.01 (0.9 to 1.13)	<0.001
North Macedonia	11.86 (8.91 to 15.88)	11.63 (8.83 to 15.52)	-0.24 (-0.3 to -0.19)	<0.001
Northern Mariana Islands	1.09 (0.82 to 1.47)	1.19 (0.9 to 1.58)	0.96 (0.86 to 1.05)	<0.001
Norway	23.82 (17.98 to 30.87)	23.97 (17.99 to 31.07)	0.09 (-0.01 to 0.19)	0.089
Oman	3.46 (2.46 to 4.8)	3.27 (2.32 to 4.66)	-0.6 (-0.94 to -0.25)	0.001
Pakistan	3.72 (2.72 to 5.04)	3.95 (2.9 to 5.31)	0.73 (0.66 to 0.81)	<0.001
Palau	1.04 (0.79 to 1.39)	1.17 (0.89 to 1.56)	1.26 (1.15 to 1.37)	<0.001
Palestine	3.58 (2.49 to 5)	3.99 (2.79 to 5.45)	1.21 (1.14 to 1.28)	<0.001
Panama	3.06 (2.29 to 4.01)	3.31 (2.53 to 4.3)	0.99 (0.83 to 1.16)	<0.001
Papua New Guinea	0.7 (0.52 to 0.97)	0.8 (0.59 to 1.08)	1.4 (1.33 to 1.48)	<0.001
Paraguay	7.67 (6.01 to 9.69)	7.01 (5.45 to 9.06)	-1.01 (-1.15 to -0.86)	<0.001
Peru	3.17 (2.39 to 4.27)	3.61 (2.73 to 4.72)	1.5 (1.27 to 1.74)	<0.001
Philippines	0.75 (0.55 to 0.99)	0.87 (0.66 to 1.15)	1.8 (1.68 to 1.91)	<0.001
Poland	15.34 (11.83 to 19.81)	13.98 (10.87 to 18)	-1.03 (-1.06 to -1)	<0.001
Portugal	8.27 (6.98 to 9.67)	8.28 (7.03 to 9.54)	0.04 (-0.06 to 0.13)	0.44
Puerto Rico	3.07 (2.46 to 3.77)	3.68 (2.85 to 4.73)	2.04 (1.63 to 2.45)	<0.001
Qatar	4.86 (3.4 to 6.95)	5.2 (3.68 to 7.49)	0.77 (0.68 to 0.86)	<0.001
Republic of Korea	5.9 (5.17 to 6.65)	6.45 (5.64 to 7.27)	1 (0.95 to 1.05)	<0.001
Republic of Moldova	4.68 (3.81 to 5.68)	5.15 (4.21 to 6.25)	1.07 (1.02 to 1.13)	<0.001
Romania	4.62 (3.89 to 5.54)	5.42 (4.56 to 6.47)	1.75 (1.68 to 1.82)	<0.001
Russian Federation	13.73 (10.71 to 17.84)	13.17 (10.45 to 16.67)	-0.43 (-0.52 to -0.33)	<0.001
Rwanda	1.83 (1.36 to 2.43)	1.8 (1.34 to 2.41)	-0.16 (-0.31 to -0.01)	0.04
Saint Kitts and Nevis	3.45 (2.5 to 4.66)	4.24 (3.09 to 5.93)	2.26 (2 to 2.53)	<0.001
Saint Lucia	3.12 (2.26 to 4.32)	3.67 (2.63 to 5)	1.91 (1.8 to 2.01)	<0.001
Saint Vincent and the Grenadines	3.53 (2.55 to 4.88)	4.07 (3.03 to 5.56)	1.6 (1.38 to 1.82)	<0.001

Samoa	0.74 (0.55 to 1.01)	0.91 (0.68 to 1.2)	2.23 (2.08 to 2.38)	<0.001
San Marino	9.5 (7.35 to 12.33)	9.66 (7.24 to 12.53)	0.24 (0.13 to 0.34)	<0.001
Sao Tome and Principe	1.63 (1.19 to 2.29)	2.14 (1.61 to 2.96)	3.19 (2.68 to 3.69)	<0.001
Saudi Arabia	2.69 (1.97 to 3.63)	2.98 (2.21 to 4.14)	1.15 (0.98 to 1.31)	<0.001
Senegal	1.93 (1.41 to 2.58)	2.21 (1.65 to 2.89)	1.59 (1.36 to 1.81)	<0.001
Serbia	11.84 (9.08 to 15.37)	12.17 (9.28 to 15.8)	0.35 (0.26 to 0.44)	<0.001
Seychelles	0.66 (0.49 to 0.89)	0.75 (0.56 to 1)	1.25 (1.11 to 1.39)	<0.001
Sierra Leone	1.76 (1.28 to 2.42)	2.06 (1.53 to 2.71)	1.78 (1.49 to 2.08)	<0.001
Singapore	2.41 (1.73 to 3.4)	2.41 (1.76 to 3.26)	-0.07 (-0.18 to 0.05)	0.224
Slovakia	7.48 (5.86 to 9.66)	7.86 (6.14 to 10.04)	0.51 (0.46 to 0.56)	<0.001
Slovenia	11.22 (8.74 to 14.49)	11.1 (8.66 to 14.4)	-0.11 (-0.12 to -0.1)	<0.001
Solomon Islands	0.76 (0.56 to 1.03)	0.83 (0.62 to 1.13)	0.99 (0.94 to 1.04)	<0.001
Somalia	1.31 (0.96 to 1.76)	1.3 (0.95 to 1.75)	-0.08 (-0.15 to -0.01)	0.038
South Africa	2.75 (2.05 to 3.75)	2.89 (2.17 to 3.87)	0.7 (0.5 to 0.91)	<0.001
South Sudan	1.75 (1.31 to 2.34)	1.78 (1.34 to 2.34)	0.17 (0.09 to 0.26)	0.001
Spain	11.11 (10.37 to 11.88)	12.13 (11.08 to 13.34)	0.96 (0.94 to 0.98)	<0.001
Sri Lanka	1.12 (0.89 to 1.4)	1.26 (1.02 to 1.58)	1.32 (1.24 to 1.41)	<0.001
Sudan	3.16 (2.21 to 4.63)	3.5 (2.45 to 5.04)	1.15 (1.12 to 1.18)	<0.001
Suriname	3.33 (2.45 to 4.52)	3.89 (2.87 to 5.29)	1.82 (1.71 to 1.93)	<0.001
Sweden	27.95 (23.15 to 33.56)	29.53 (24.5 to 35.21)	0.56 (0.42 to 0.7)	<0.001
Switzerland	11.65 (9.27 to 14.64)	11.02 (8.68 to 13.96)	-0.52 (-0.63 to -0.41)	<0.001
Syrian Arab Republic	3.33 (2.33 to 4.86)	4.35 (3.08 to 5.9)	3.23 (2.75 to 3.7)	<0.001
Taiwan (Province of China)	1.38 (1.1 to 1.72)	1.62 (1.3 to 2.01)	1.86 (1.66 to 2.06)	<0.001
Tajikistan	8.89 (6.64 to 11.79)	9.68 (7.31 to 12.87)	0.95 (0.74 to 1.17)	<0.001
Thailand	0.43 (0.31 to 0.59)	0.49 (0.36 to 0.65)	1.4 (1.3 to 1.5)	<0.001
Timor-Leste	0.57 (0.42 to 0.75)	0.62 (0.45 to 0.83)	1.02 (0.97 to 1.08)	<0.001
Togo	2.02 (1.5 to 2.7)	2.29 (1.72 to 3.03)	1.45 (1.18 to 1.73)	<0.001
Tokelau	0.77 (0.57 to 1.02)	0.98 (0.73 to 1.32)	2.79 (2.59 to 2.98)	<0.001
Tonga	0.9 (0.67 to 1.18)	1.01 (0.76 to 1.34)	1.31 (1.16 to 1.45)	<0.001
Trinidad and Tobago	4.1 (3 to 5.69)	4.74 (3.46 to 6.36)	1.64 (1.41 to 1.88)	<0.001
Tunisia	3.45 (2.43 to 4.96)	3.51 (2.51 to 4.87)	0.18 (0.15 to 0.22)	<0.001
Turkey	5.22 (4.21 to 6.5)	6.38 (4.66 to 8.72)	2.34 (2.12 to 2.55)	<0.001
Turkmenistan	11.44 (8.56 to 15.01)	12.11 (9.15 to 16.25)	0.71 (0.56 to 0.86)	<0.001
Tuvalu	0.94 (0.71 to 1.22)	1.04 (0.79 to 1.38)	1.1 (1.03 to 1.17)	<0.001
Uganda	1.66 (1.23 to 2.22)	1.84 (1.35 to 2.48)	1.15 (1.05 to 1.25)	<0.001
Ukraine	11.06 (8.42 to 14.47)	11.17 (8.38 to 14.66)	0.14 (-0.05 to 0.33)	0.145
United Arab Emirates	5.96 (4.2 to 8.25)	6.18 (4.28 to 8.73)	0.32 (0.15 to 0.49)	<0.001
United Kingdom	14.73 (11.57 to 18.49)	14.42 (11.28 to 18.23)	-0.2 (-0.31 to -0.09)	<0.001
United Republic of Tanzania	1.58 (1.17 to 2.11)	1.82 (1.31 to 2.41)	1.61 (1.49 to 1.73)	<0.001
United States of America	27.92 (23.12 to 33.46)	28.87 (23.74 to 34.82)	0.39 (0.36 to 0.41)	<0.001

United States Virgin Islands	3.91 (2.77 to 5.4)	4.21 (3.01 to 5.83)	0.8 (0.59 to 1.02)	<0.001
Uruguay	1.45 (1.1 to 1.95)	1.59 (1.21 to 2.1)	1.06 (0.93 to 1.19)	<0.001
Uzbekistan	9.64 (7.13 to 13.08)	10.89 (8.15 to 14.7)	1.4 (1.22 to 1.58)	<0.001
Vanuatu	0.82 (0.6 to 1.09)	0.9 (0.66 to 1.21)	0.95 (0.84 to 1.05)	<0.001
Venezuela (Bolivarian Republic of)	2.87 (2.18 to 3.71)	3.23 (2.44 to 4.19)	1.31 (1.17 to 1.46)	<0.001
Viet Nam	0.84 (0.61 to 1.14)	1 (0.74 to 1.36)	2.02 (1.85 to 2.18)	<0.001
Yemen	3.06 (2.17 to 4.49)	3.12 (2.18 to 4.38)	0.2 (0.14 to 0.25)	<0.001
Zambia	2.02 (1.5 to 2.69)	1.87 (1.37 to 2.51)	-0.96 (-1.23 to -0.68)	<0.001
Zimbabwe	2.2 (1.64 to 2.97)	2.36 (1.79 to 3.11)	0.84 (0.68 to 0.99)	<0.001

Abbreviation: ASIR: age-standardize incidence rate; APC: annual percentage change; CI: confidence interval; UI: uncertainty interval

Supplementary Material Table S4. Age-specific disability adjusted life years rates (ASDALYs) of inflammatory bowel disease in patients aged 65-89 years in 2010 and 2019, stratified by countries.

Countries	2010 ASDALYs, per 100,000 (95% UI)	2019 ASDALYs, per 100,000 (95% UI)	APC (95% CI)	***p***
Afghanistan	49.66 (30.58 to 81.02)	48.25 (30.06 to 75.89)	-0.23 (-0.34 to -0.11)	0.002
Albania	48.92 (36.13 to 69.55)	51.15 (37.28 to 69.65)	0.51 (0.42 to 0.6)	<0.001
Algeria	26.08 (18.46 to 36.92)	24.86 (17.95 to 33.49)	-0.51 (-0.62 to -0.4)	<0.001
American Samoa	72.1 (56.77 to 91.21)	47.72 (33.86 to 78.25)	-4.63 (-5.52 to -3.73)	<0.001
Andorra	91.53 (62.17 to 123.4)	93.42 (60.81 to 128.87)	0.11 (-0.19 to 0.41)	0.46
Angola	61.79 (41.52 to 93.73)	60.09 (41.14 to 89.55)	-0.25 (-0.51 to 0.01)	0.055
Antigua and Barbuda	75.19 (63.35 to 95.31)	70.46 (55.61 to 93.47)	-0.64 (-1 to -0.29)	<0.001
Argentina	28.26 (25.18 to 33.04)	30.49 (26 to 36.06)	1.26 (0.73 to 1.8)	0.001
Armenia	47.45 (37.2 to 60.82)	48.32 (37.8 to 61.47)	0.24 (0.06 to 0.43)	0.016
Australia	107.31 (80.57 to 123.31)	101.7 (78.84 to 118.83)	-0.72 (-0.95 to -0.49)	<0.001
Austria	143.59 (119.32 to 174.51)	102.42 (81.07 to 138.08)	-3.64 (-4.12 to -3.15)	<0.001
Azerbaijan	34.62 (24.65 to 47.23)	35.76 (25.19 to 49.43)	0.43 (0.16 to 0.7)	0.002
Bahamas	48.83 (41.33 to 58.33)	47.31 (38.35 to 58.54)	-0.36 (-0.66 to -0.05)	0.023
Bahrain	29.18 (23.45 to 36.19)	24.2 (18.2 to 31.17)	-1.88 (-2.5 to -1.27)	<0.001
Bangladesh	68.82 (42.65 to 104.68)	58.49 (35.86 to 88.04)	-1.67 (-2.36 to -0.98)	<0.001
Barbados	55.93 (45.57 to 68.33)	54.89 (43.28 to 68.14)	-0.33 (-0.73 to 0.07)	0.105
Belarus	62.79 (50.53 to 77.7)	60.67 (47 to 78.13)	-0.51 (-0.89 to -0.13)	0.015
Belgium	142.76 (103.52 to 161.03)	121.84 (99.37 to 142.2)	-1.9 (-2.83 to -0.97)	<0.001
Belize	59.77 (49.73 to 77.24)	55.27 (44.82 to 73.96)	-0.78 (-1.38 to -0.17)	0.019
Benin	38.56 (25.1 to 57.01)	40.19 (28.37 to 55.51)	0.34 (-0.19 to 0.87)	0.204

Bermuda	48.58 (40.57 to 61.31)	43.56 (34.22 to 57.51)	-1.03 (-1.59 to -0.47)	<0.001
Bhutan	64.82 (40.24 to 144.19)	61.64 (38.25 to 135.8)	-0.5 (-0.56 to -0.44)	<0.001
Bolivia (Plurinational State of)	43.22 (32 to 56.13)	42.93 (32.23 to 56.9)	-0.09 (-0.17 to -0.02)	0.018
Bosnia and Herzegovina	69.43 (49.21 to 85.96)	77.23 (49.21 to 100.2)	1.65 (1.09 to 2.21)	<0.001
Botswana	69.82 (49.67 to 97.02)	60.49 (42.04 to 85.3)	-1.58 (-1.72 to -1.44)	<0.001
Brazil	58.87 (50.95 to 69.61)	57.07 (49.46 to 67.34)	-0.31 (-0.64 to 0.03)	0.076
Brunei Darussalam	135.61 (98.32 to 166.34)	121.24 (87.91 to 153.18)	-1.25 (-1.47 to -1.03)	<0.001
Bulgaria	52.41 (41.2 to 65.84)	54.46 (42.81 to 69.39)	0.3 (0.11 to 0.49)	0.007
Burkina Faso	58.58 (36.99 to 86.52)	57.14 (33.77 to 87.49)	-0.33 (-0.82 to 0.16)	0.187
Burundi	64.74 (40.05 to 98.88)	71.61 (42.76 to 119.72)	1.12 (0.85 to 1.39)	<0.001
C?¥te d'Ivoire	37.66 (22.99 to 56.62)	36.52 (23.82 to 54.08)	-0.41 (-0.78 to -0.03)	0.036
Cabo Verde	25.22 (18.66 to 32.55)	27.31 (20.31 to 35.67)	1.67 (-2.33 to 5.83)	0.42
Cambodia	41.5 (25.61 to 57.15)	38.15 (24.98 to 51.6)	-0.92 (-1.09 to -0.75)	<0.001
Cameroon	35.76 (20.55 to 55.05)	33.49 (19.89 to 51.08)	-0.75 (-1 to -0.49)	<0.001
Canada	151.05 (121.76 to 185.01)	129.76 (102.36 to 166.28)	-1.71 (-1.98 to -1.45)	<0.001
Central African Republic	86.53 (52.06 to 144.55)	82.83 (49.19 to 138.11)	-0.46 (-0.59 to -0.32)	<0.001
Chad	56.67 (35.49 to 89.05)	51.6 (34.65 to 75.89)	-1.04 (-1.25 to -0.84)	<0.001
Chile	33.72 (28.26 to 45.03)	35.62 (29.92 to 45.51)	0.93 (0.25 to 1.62)	0.008
China	50.57 (41.58 to 57.61)	40.13 (33.09 to 47.95)	-2.56 (-2.71 to -2.42)	<0.001
Colombia	42.12 (35.75 to 50.33)	39.42 (30.85 to 49.41)	-0.76 (-0.94 to -0.58)	<0.001
Comoros	80.34 (47.74 to 131.07)	76.81 (47.53 to 126.18)	-0.52 (-0.77 to -0.27)	<0.001
Congo	74.74 (49.32 to 109.47)	64.36 (45.65 to 91.27)	-1.63 (-1.8 to -1.45)	<0.001
Cook Islands	69.04 (48.53 to 94.6)	63.42 (42.78 to 88.57)	-1.04 (-1.23 to -0.86)	<0.001
Costa Rica	29.92 (24.18 to 36.85)	29.58 (22.64 to 37.51)	0.18 (-0.46 to 0.83)	0.53
Croatia	73.28 (61.63 to 87.3)	73.08 (57.57 to 90.46)	-0.09 (-0.36 to 0.18)	0.46
Cuba	29.37 (24.59 to 35.02)	29.96 (23.76 to 37.66)	0.71 (0.14 to 1.3)	0.021
Cyprus	114.78 (86.9 to 141.97)	96.22 (75.23 to 119.03)	-1.83 (-2.4 to -1.26)	<0.001
Czechia	91.45 (70.64 to 106.13)	89.85 (71.85 to 110.72)	-0.13 (-0.36 to 0.09)	0.253
Democratic People's Republic of Korea	42.63 (30.6 to 58.94)	42.02 (29.41 to 57.39)	-0.15 (-0.22 to -0.09)	<0.001
Democratic Republic of the Congo	55.28 (37.92 to 79.05)	53.39 (36.19 to 77.87)	-0.33 (-0.57 to -0.1)	0.006
Denmark	106.12 (90.94 to 123.08)	93.38 (79.13 to 110.32)	-1.36 (-1.61 to -1.1)	<0.001
Djibouti	56.18 (33.66 to 100.36)	54.04 (34.73 to 89.34)	-0.48 (-0.66 to -0.3)	<0.001
Dominica	76.93 (59.67 to 98.48)	75.36 (55.61 to 103.03)	-0.34 (-0.62 to -0.05)	0.027
Dominican Republic	25.19 (19.44 to 31.94)	25.78 (19.45 to 33.45)	0.41 (-0.01 to 0.82)	0.054
Ecuador	25.61 (21.42 to 30.44)	27.64 (21.29 to 34.69)	1 (0.58 to 1.41)	<0.001
Egypt	30.53 (20.39 to 50.11)	31.01 (19.44 to 48.13)	0.26 (0.03 to 0.49)	0.034
El Salvador	23.72 (19.26 to 29.38)	24.12 (18.41 to 31.04)	0.13 (-0.27 to 0.53)	0.535
Equatorial Guinea	58.26 (33.93 to 91.19)	56.74 (35.22 to 89.4)	-0.16 (-0.36 to 0.05)	0.115
Eritrea	69.69 (41.75 to 113.85)	70.65 (44.85 to 107.9)	0.17 (0.1 to 0.25)	<0.001
Estonia	51.58 (42.09 to 63.26)	57.83 (44.35 to 73.74)	1.35 (1.11 to 1.6)	<0.001

Eswatini	67.87 (46.12 to 97.75)	57.3 (41.46 to 77.62)	-1.85 (-1.94 to -1.76)	<0.001
Ethiopia	70.39 (42.35 to 103.83)	76.23 (43.77 to 115.32)	0.91 (0.78 to 1.04)	<0.001
Fiji	23.67 (19.31 to 28.83)	23.01 (16.45 to 30.94)	-0.36 (-0.47 to -0.24)	<0.001
Finland	78.91 (63.61 to 96.81)	85.06 (66.22 to 108.12)	0.92 (0.5 to 1.34)	<0.001
France	134.82 (92.89 to 150.98)	135.47 (85.36 to 162.22)	0 (-0.49 to 0.49)	0.997
Gabon	60.58 (42.81 to 88.07)	54.52 (37.6 to 79.85)	-1.04 (-1.23 to -0.86)	<0.001
Gambia	41.7 (28.31 to 57.68)	41.8 (28.39 to 57.9)	0.61 (0.04 to 1.19)	0.039
Georgia	44.29 (33.64 to 56.76)	56.87 (41.29 to 73.52)	2.69 (2.46 to 2.91)	<0.001
Germany	226.03 (186.34 to 251.63)	243.57 (190.5 to 278.51)	0.78 (0.24 to 1.31)	0.004
Ghana	47.76 (28.29 to 71.01)	49.65 (29.04 to 77.08)	0.36 (0 to 0.73)	0.047
Greece	33.67 (26.63 to 42.81)	36.87 (30.39 to 44.35)	0.92 (0.46 to 1.39)	<0.001
Greenland	109.1 (78.01 to 136.32)	109.32 (72.55 to 138.91)	0.04 (-0.16 to 0.25)	0.683
Grenada	117.81 (97 to 143.41)	122.02 (98.81 to 152.25)	0.56 (-0.71 to 1.84)	0.388
Guam	23.18 (17.17 to 30.17)	16.29 (11.69 to 22.68)	-3.68 (-4.56 to -2.79)	<0.001
Guatemala	31.88 (25.54 to 38.98)	34.7 (26.14 to 43.05)	0.69 (-0.05 to 1.44)	0.068
Guinea	37.15 (21.54 to 63.45)	34.15 (20.67 to 52.08)	-0.92 (-1.03 to -0.8)	<0.001
Guinea-Bissau	54.49 (36.05 to 75.91)	49.84 (33.52 to 71.61)	-0.98 (-1.05 to -0.9)	<0.001
Guyana	80.68 (67.51 to 101.95)	82.17 (61.75 to 108.06)	0.34 (-0.08 to 0.75)	0.097
Haiti	100.57 (58.04 to 168.19)	92.99 (54.85 to 152.06)	-0.73 (-0.85 to -0.62)	<0.001
Honduras	87.52 (54.21 to 121.61)	91.34 (56.72 to 126)	0.66 (0.27 to 1.04)	0.001
Hungary	137.27 (112.23 to 166.26)	126.29 (98.54 to 158.47)	-0.86 (-1.14 to -0.57)	<0.001
Iceland	106.61 (88.45 to 127.73)	100.9 (80.1 to 123.12)	-0.9 (-1.29 to -0.51)	0.001
India	47.76 (37.59 to 63.88)	43.79 (32.9 to 55.95)	-1 (-1.28 to -0.72)	<0.001
Indonesia	62.4 (38.86 to 79.14)	58.95 (35.88 to 75.82)	-0.49 (-0.9 to -0.07)	0.023
Iran (Islamic Republic of)	26.54 (19.75 to 33.06)	31.88 (19.37 to 40.09)	2.11 (1.84 to 2.38)	<0.001
Iraq	24.17 (17.92 to 34.72)	21.46 (15.69 to 31.5)	-1.31 (-1.61 to -1)	<0.001
Ireland	107.65 (90.12 to 123.85)	86.21 (71.07 to 105.86)	-2.47 (-2.69 to -2.26)	<0.001
Israel	57.63 (49.24 to 70.2)	47.92 (39.5 to 62.58)	-2.11 (-2.48 to -1.73)	<0.001
Italy	142.12 (106.59 to 165.85)	129.96 (101.76 to 153.27)	-1.48 (-2.12 to -0.84)	0.001
Jamaica	30.94 (24.96 to 40.95)	29.43 (21.92 to 41.01)	-0.59 (-0.89 to -0.3)	<0.001
Japan	31.91 (24.36 to 41.54)	31.22 (23.65 to 40.4)	-0.25 (-0.33 to -0.17)	<0.001
Jordan	52.08 (40.14 to 67.83)	48.14 (36.59 to 63.54)	-0.73 (-1.13 to -0.33)	<0.001
Kazakhstan	76.39 (61.27 to 93.5)	62.08 (47.64 to 78.38)	-2.63 (-3.7 to -1.55)	<0.001
Kenya	83.21 (49.54 to 129.93)	87.81 (51.79 to 130.59)	0.55 (0.33 to 0.77)	<0.001
Kiribati	95.73 (58.57 to 163.42)	82.37 (53.06 to 132.52)	-1.62 (-1.85 to -1.39)	<0.001
Kuwait	29.13 (23.65 to 35.39)	28.01 (21.58 to 36.81)	-0.32 (-1.23 to 0.61)	0.5
Kyrgyzstan	35.75 (28 to 45.55)	35.53 (26.94 to 46.59)	-0.17 (-0.58 to 0.25)	0.425
Lao People's Democratic Republic	28.63 (15.38 to 50.54)	26.42 (14.25 to 44.86)	-0.89 (-0.96 to -0.82)	<0.001
Latvia	65.43 (53.1 to 80.02)	64.33 (50.76 to 79.81)	-0.17 (-0.37 to 0.04)	0.105
Lebanon	25.08 (17.03 to 33.78)	25.53 (16.2 to 36.32)	0.17 (0.1 to 0.25)	0.001

Lesotho	72.26 (50.5 to 99.69)	68.55 (48.5 to 95.89)	-0.56 (-0.79 to -0.33)	<0.001
Liberia	39.25 (22.24 to 60.2)	37.92 (24.83 to 54.44)	-0.49 (-1.28 to 0.32)	0.237
Libya	28.6 (20.81 to 38.82)	28.74 (19.54 to 40.96)	0.06 (-0.55 to 0.68)	0.846
Lithuania	60.66 (49.02 to 73.17)	60.48 (47.78 to 74.68)	-0.12 (-0.69 to 0.47)	0.66
Luxembourg	158.52 (131.93 to 184.96)	137.9 (107.78 to 164.31)	-1.62 (-2.06 to -1.18)	<0.001
Madagascar	51.44 (32.23 to 81.57)	56.18 (36.01 to 83.8)	0.97 (0.83 to 1.11)	<0.001
Malawi	61.38 (41.16 to 89.93)	65.55 (42.7 to 102.14)	0.7 (0.23 to 1.17)	0.003
Malaysia	20.39 (15.54 to 28.81)	18.56 (12.55 to 26.82)	-1.3 (-2.76 to 0.18)	0.086
Maldives	35.08 (26.49 to 46.62)	32.98 (23.67 to 45.02)	-0.62 (-0.7 to -0.55)	<0.001
Mali	40.66 (25.34 to 64.16)	39.55 (24.38 to 59)	-0.12 (-0.36 to 0.13)	0.313
Malta	76.69 (62.53 to 89.31)	71.71 (56.72 to 86.25)	-1.12 (-1.52 to -0.72)	<0.001
Marshall Islands	71.68 (42.26 to 106.68)	58.78 (34.62 to 93.24)	-2.21 (-2.37 to -2.05)	<0.001
Mauritania	47.44 (32.51 to 68.48)	40.3 (27.64 to 56.16)	-1.71 (-1.89 to -1.53)	<0.001
Mauritius	22.12 (18.22 to 25.81)	21.88 (16.29 to 28.2)	0.3 (-1.23 to 1.85)	0.702
Mexico	52.76 (45.04 to 60.2)	56.9 (46.69 to 67.17)	0.79 (0.42 to 1.16)	<0.001
Micronesia (Federated States of)	73.52 (44.54 to 110.1)	60.44 (37.73 to 92.13)	-2.19 (-2.3 to -2.08)	<0.001
Monaco	64.92 (50.68 to 79.75)	61.43 (46.66 to 79.22)	-0.62 (-0.65 to -0.58)	<0.001
Mongolia	59.55 (45.4 to 77.96)	53.27 (39.57 to 69.11)	-1.32 (-1.53 to -1.1)	<0.001
Montenegro	64.98 (50.67 to 82.59)	60.45 (46.05 to 77.65)	-0.76 (-0.82 to -0.7)	<0.001
Morocco	25.03 (17.42 to 36.39)	25.74 (18.62 to 33.74)	0.47 (-0.23 to 1.17)	0.188
Mozambique	77.03 (51.36 to 113.71)	86.79 (56.04 to 128.5)	1.45 (0.94 to 1.97)	<0.001
Myanmar	15.15 (10.41 to 20.94)	13.06 (9.6 to 17.52)	-1.64 (-1.73 to -1.54)	<0.001
Namibia	69 (51.79 to 91.36)	65.63 (47.81 to 89.73)	-0.49 (-0.72 to -0.27)	<0.001
Nauru	73.1 (47.07 to 106.95)	59.34 (37.48 to 90.44)	-2.32 (-2.75 to -1.88)	<0.001
Nepal	64.54 (38.21 to 117.83)	62.84 (39.46 to 107.7)	-0.18 (-0.3 to -0.06)	0.01
Netherlands	225.31 (185.09 to 250.12)	201.31 (164.27 to 231.71)	-1.3 (-1.6 to -1)	<0.001
New Zealand	67.57 (56.48 to 81.19)	65.66 (54.25 to 79.43)	-0.4 (-0.95 to 0.15)	0.15
Nicaragua	26.09 (21.43 to 31.37)	27.93 (21.84 to 34.46)	1.11 (0.73 to 1.48)	<0.001
Niger	40.58 (23.53 to 65.66)	44.18 (27.31 to 69.74)	0.93 (0.62 to 1.24)	<0.001
Nigeria	55.49 (27.54 to 91.39)	51.5 (28.46 to 81.44)	-0.83 (-0.98 to -0.67)	<0.001
Niue	67.88 (43.09 to 97.82)	60.57 (36.33 to 89.33)	-1.22 (-1.29 to -1.14)	<0.001
North Macedonia	40.99 (30.87 to 54.26)	41.68 (31.05 to 56.2)	0.29 (0.15 to 0.42)	0.001
Northern Mariana Islands	58.42 (44.87 to 76.26)	32.2 (21.37 to 60.59)	-6.58 (-7.69 to -5.46)	<0.001
Norway	126.57 (97.56 to 163.26)	121.22 (91.41 to 158.88)	-0.56 (-0.67 to -0.45)	<0.001
Oman	37.57 (22.72 to 60.77)	32.1 (20.71 to 47.42)	-1.86 (-2.51 to -1.21)	<0.001
Pakistan	68.09 (41.31 to 117.06)	61.87 (40.58 to 98.09)	-1.05 (-1.2 to -0.9)	<0.001
Palau	55.81 (31.43 to 87.94)	44.67 (23.3 to 69.59)	-2.62 (-2.84 to -2.39)	<0.001
Palestine	34.15 (27.22 to 42.48)	30.07 (22.98 to 40)	-1.44 (-1.97 to -0.91)	<0.001
Panama	50.41 (42.31 to 63.12)	57.56 (44.36 to 72.06)	1.48 (-0.06 to 3.03)	0.059
Papua New Guinea	35.23 (21.76 to 55.16)	31.92 (19.69 to 48.86)	-1.17 (-1.56 to -0.78)	<0.001

Paraguay	31.64 (25.36 to 38.66)	33.58 (24.42 to 43.08)	0.65 (0.13 to 1.18)	0.015
Peru	29.48 (22.99 to 37.83)	26.25 (19.28 to 35.01)	-1.31 (-1.56 to -1.06)	<0.001
Philippines	19.64 (16.25 to 21.85)	16.95 (13.89 to 21.42)	-1.66 (-2.12 to -1.21)	<0.001
Poland	91.68 (75.51 to 108.69)	92.28 (73.92 to 112.07)	-0.05 (-0.49 to 0.39)	0.822
Portugal	128.89 (72.45 to 147.32)	120.73 (69 to 143.58)	-0.87 (-1.39 to -0.36)	0.001
Puerto Rico	34.5 (28.83 to 44.66)	30.37 (22.67 to 44.42)	-1.47 (-2.29 to -0.64)	0.001
Qatar	80.04 (37.36 to 112.62)	48.44 (26.26 to 72.05)	-5.62 (-5.86 to -5.37)	<0.001
Republic of Korea	42.85 (33.95 to 51.07)	36.7 (29.45 to 46.51)	-1.75 (-1.98 to -1.51)	<0.001
Republic of Moldova	38.08 (31.53 to 47.16)	40.16 (32.34 to 49.47)	0.85 (-0.28 to 1.99)	0.143
Romania	30.49 (25.09 to 37.58)	33.08 (26.33 to 41.03)	0.82 (0.22 to 1.42)	0.008
Russian Federation	73.61 (63.85 to 85.74)	77.17 (63.52 to 91.74)	0.62 (-0.08 to 1.32)	0.085
Rwanda	60.13 (37.15 to 92.09)	60.87 (37.57 to 92.05)	0.19 (0.12 to 0.26)	<0.001
Saint Kitts and Nevis	140.67 (114.06 to 184.26)	149.61 (115.64 to 211.13)	0.78 (-0.75 to 2.34)	0.319
Saint Lucia	50.58 (42.12 to 64.02)	59.16 (46.73 to 75.75)	2.22 (1.71 to 2.74)	<0.001
Saint Vincent and the Grenadines	100.3 (84.53 to 123.08)	105.6 (84.01 to 134.69)	0.71 (0.35 to 1.08)	0.002
Samoa	62.47 (43.3 to 88.43)	58.12 (42.17 to 79.16)	-0.82 (-0.88 to -0.75)	<0.001
San Marino	62.68 (44.28 to 85.17)	60.71 (42.35 to 82.31)	-0.36 (-0.45 to -0.27)	<0.001
Sao Tome and Principe	41.52 (26.36 to 72.54)	32.81 (21.6 to 52.42)	-2.49 (-2.68 to -2.3)	<0.001
Saudi Arabia	24.79 (19.1 to 34.34)	21.8 (15.71 to 29.49)	-1.51 (-1.68 to -1.34)	<0.001
Senegal	39.25 (25.03 to 63.06)	39.92 (25.03 to 59.73)	0.04 (-0.32 to 0.41)	0.826
Serbia	123.08 (89.21 to 145.83)	111.25 (79.83 to 139.34)	-1.24 (-1.56 to -0.93)	<0.001
Seychelles	25.83 (18.41 to 34.42)	23.17 (16.46 to 31.88)	-1.13 (-1.26 to -1.01)	<0.001
Sierra Leone	37.6 (24.42 to 53.82)	36.59 (23.51 to 52.01)	-0.38 (-0.92 to 0.15)	0.161
Singapore	13.28 (10.79 to 17.65)	12.21 (9.46 to 16.57)	-0.94 (-1.35 to -0.54)	<0.001
Slovakia	105.15 (79.68 to 122.27)	97.6 (70.94 to 123.91)	-0.87 (-1.17 to -0.57)	<0.001
Slovenia	85.55 (64.86 to 102.74)	75.43 (57.8 to 95.54)	-1.6 (-2.03 to -1.17)	<0.001
Solomon Islands	40.16 (25.75 to 61.18)	38.05 (24.89 to 55.17)	-0.64 (-0.92 to -0.36)	<0.001
Somalia	46.94 (26.28 to 78.92)	44.26 (26.08 to 71.68)	-0.59 (-0.76 to -0.41)	<0.001
South Africa	50.21 (39.44 to 55.09)	41.01 (31.26 to 48.58)	-1.8 (-2.26 to -1.33)	<0.001
South Sudan	68.52 (35.22 to 158.7)	66.01 (32.77 to 142.37)	-0.34 (-0.41 to -0.27)	<0.001
Spain	63.2 (53 to 79.14)	63.13 (51.65 to 80.01)	-0.06 (-0.37 to 0.24)	0.683
Sri Lanka	13 (10.7 to 16.1)	11.44 (8.35 to 15.7)	-1.48 (-1.61 to -1.34)	<0.001
Sudan	32.48 (17.51 to 58.76)	33.61 (20.32 to 55.17)	0.36 (0.21 to 0.5)	<0.001
Suriname	65.46 (52.97 to 83.81)	63.94 (48.06 to 84.51)	0.08 (-0.26 to 0.42)	0.613
Sweden	112.8 (91.57 to 138.3)	97.94 (75.51 to 126.11)	-1.6 (-1.87 to -1.33)	<0.001
Switzerland	106.65 (89.67 to 124.09)	88.96 (73.62 to 107.92)	-1.88 (-2.48 to -1.28)	<0.001
Syrian Arab Republic	49.9 (28.41 to 65.33)	46.78 (24.8 to 67.23)	-0.82 (-1 to -0.64)	<0.001
Taiwan (Province of China)	45.99 (38.39 to 68.22)	56.44 (44.2 to 73.61)	2.12 (1.73 to 2.5)	<0.001
Tajikistan	46.17 (35.98 to 60.08)	45.8 (34.12 to 59.72)	-0.08 (-0.31 to 0.15)	0.503
Thailand	11.53 (8.91 to 16.29)	11.88 (8.45 to 16.3)	0.91 (0.26 to 1.58)	0.013

Timor-Leste	39.81 (19.47 to 83.32)	38.41 (20.81 to 69.85)	-0.6 (-0.85 to -0.34)	0.001
Togo	39.23 (27.03 to 55.56)	41.33 (26.8 to 58.9)	0.67 (0.05 to 1.29)	0.033
Tokelau	61.99 (44.35 to 86.6)	51.71 (37.01 to 71.17)	-1.89 (-2.21 to -1.58)	<0.001
Tonga	77.97 (55.92 to 105.91)	73.83 (53.62 to 101.19)	-0.68 (-0.78 to -0.59)	<0.001
Trinidad and Tobago	37.09 (28.38 to 44.73)	34.69 (25.72 to 44.54)	-0.08 (-0.88 to 0.73)	0.827
Tunisia	25.07 (18.28 to 33.08)	24.18 (17.47 to 32.27)	-0.4 (-0.47 to -0.33)	<0.001
Turkey	61.94 (50.05 to 82.63)	56.37 (42.54 to 81.1)	-1.15 (-1.56 to -0.73)	<0.001
Turkmenistan	41.57 (32.87 to 52.97)	46.86 (35.53 to 61.08)	1.33 (1.03 to 1.62)	<0.001
Tuvalu	63.64 (39.52 to 92.39)	56.06 (36.83 to 82.77)	-1.4 (-1.53 to -1.27)	<0.001
Uganda	57.68 (38.11 to 86.16)	60.89 (38.07 to 94.27)	0.65 (0.54 to 0.76)	<0.001
Ukraine	45.37 (36.8 to 56.33)	47.76 (37.62 to 59.8)	0.64 (0.15 to 1.13)	0.01
United Arab Emirates	45.23 (25.88 to 87.9)	33.56 (20.84 to 59.27)	-3.81 (-5.06 to -2.55)	<0.001
United Kingdom	180.18 (154.95 to 199.6)	166.77 (145.44 to 186.46)	-0.84 (-1.08 to -0.6)	<0.001
United Republic of Tanzania	57.64 (39.52 to 83.34)	58.12 (38.27 to 91.83)	0.06 (-0.06 to 0.18)	0.333
United States of America	156.29 (125.36 to 176.06)	150.31 (123.45 to 169.36)	-0.51 (-0.75 to -0.27)	<0.001
United States Virgin Islands	50.87 (40.38 to 63.83)	50.39 (38.74 to 64.86)	-0.1 (-0.16 to -0.03)	0.003
Uruguay	49.91 (40.1 to 56.68)	47.94 (40.16 to 55.8)	-0.89 (-1.48 to -0.29)	0.009
Uzbekistan	39.58 (29.68 to 53.8)	41.06 (30.07 to 55.82)	0.61 (0.34 to 0.89)	0.001
Vanuatu	71.56 (42.24 to 117.23)	66.72 (41.75 to 104.28)	-0.76 (-0.83 to -0.69)	<0.001
Venezuela (Bolivarian Republic of)	25.22 (19.93 to 32.24)	25.04 (18.91 to 32.53)	0.1 (-0.13 to 0.32)	0.355
Viet Nam	31.14 (23.2 to 41.71)	27.73 (18.79 to 38.93)	-1.2 (-1.29 to -1.11)	<0.001
Yemen	41.87 (25.39 to 71.27)	41.43 (26.92 to 64.22)	-0.28 (-0.45 to -0.1)	0.006
Zambia	73.61 (51.82 to 105.75)	65.42 (46.44 to 92.65)	-1.27 (-1.52 to -1.02)	<0.001
Zimbabwe	60.02 (43.7 to 85.31)	56.34 (32.82 to 88)	-0.65 (-0.89 to -0.41)	<0.001

Abbreviation: ASDALYs: age-standardize disability-adjusted life year rate; APC: annual percentage change; CI: confidence interval; UI: uncertainty interval
